# Interplay between tumor mutation burden and the tumor microenvironment predicts the prognosis of pan-cancer anti-PD-1/PD-L1 therapy

**DOI:** 10.3389/fimmu.2025.1557461

**Published:** 2025-07-24

**Authors:** Wuyuan Liao, Xinwei Zhou, Hansen Lin, Zihao Feng, Xinyan Chen, Yuhang Chen, Minyu Chen, Mingjie Lin, Gaosheng Yao, Jinwei Chen, Haoqian Feng, Yinghan Wang, Zhiping Tan, Youyan Tan, Jun Lu, Pengju Li, Jinhuan Wei, Li Luo, Liangmin Fu

**Affiliations:** ^1^ Department of Urology, The First Affiliated Hospital of Sun Yat-sen University, Guangzhou, Guangdong, China; ^2^ Institute of Precision Medicine, The First Affiliated Hospital, Sun Yat-sen University, Guangzhou, Guangdong, China; ^3^ Department of Urology, Cancer Hospital Chinese Academy of Medical Sciences, Shenzhen Center, Shenzhen, China; ^4^ Guangdong Pharmaceutical University, Guangzhou, Guangdong, China; ^5^ The Seventh Affiliated Hospital, Sun Yat-sen University, Shenzhen, Guangdong, China; ^6^ Department of Oncology, The First Affiliated Hospital of Sun Yat-sen University, Guangzhou, Guangdong, China; ^7^ Department of Urology, The Second Xiangya Hospital of Central South University, Changsha, Hunan, China; ^8^ Uro-Oncology Institute of Central South University, Changsha, Hunan, China; ^9^ Key Laboratory of Diabetes Immunology (Central South University), Ministry of Education, National Clinical Research Center for Metabolic Disease, Changsha, China

**Keywords:** immune checkpoint inhibitor, tumor mutation burden, tumor microenvironment, pan-cancer, biomarker

## Abstract

**Introduction:**

Immune checkpoint inhibitors (ICIs) have revolutionized the treatment landscape for advanced cancers, yet their efficacy remains heterogeneous among patients. Tumor mutation burden (TMB) has been extensively explored as a potential biomarker for predicting ICI response. However, its application is limited by several factors, including inconsistent predictive power across different tumor types and the lack of a clear relationship with overall survival (OS). This study aimed to explore the complex interplay between TMB and the tumor microenvironment (TME) and to identify novel predictive biomarkers that can enhance the precision of ICI therapy across multiple cancer types.

**Methods:**

We systematically collected and analyzed genomic and clinical data from patients receiving anti-PD-1/PD-L1 immunotherapy across multiple cohorts. Our dataset included information from The Cancer Genome Atlas (TCGA) pan-cancer database and various ICI clinical trials. We first screened immunosuppression-related genes (ISRGs) that might interfere with TMB's predictive role by analyzing the survival data and gene expression profiles of patients. Using LASSO regression and multivariable Cox proportional hazards analysis, we constructed a risk model based on these ISRGs. The model's predictive ability was rigorously validated in multiple independent cohorts. Additionally, we employed algorithms such as CIBERSORT and ESTIMATE to assess the correlation between the risk score and TME components. To further explore the therapeutic implications of our findings, we focused on RPLP0, a ribosomal protein that emerged as a robust biomarker in our model. We investigated its expression in tumor tissues and evaluated the impact of its knockdown on immunotherapeutic efficacy using in vitro and in vivo experiments.

**Results:**

Our comprehensive analysis revealed that the predictive power of TMB varies significantly across different cancer types and is highly dependent on its interaction with the TME. In tumors with a favorable immune microenvironment, characterized by high CD8+ T cell infiltration and M1 macrophage presence, TMB maintained its predictive ability. However, in immunosuppressive microenvironments, TMB alone failed to accurately predict patient outcomes. We identified 304 ISRGs and developed a 10-gene risk signature that demonstrated reliable prognostic predictive ability in both ICI cohorts and TCGA pan-cancer. The risk score derived from this model was significantly associated with stromal components and an immunosuppressive TME, characterized by elevated levels of M0 macrophages and activated mast cells. Notably, RPLP0 was identified as the most robust predictive marker during model building. We demonstrated its abnormal overexpression in tumor tissues and further showed that intratumoral RPLP0 knockdown in a subcutaneous bladder cancer model could enhance the efficacy of immunotherapy. The combination of RPLP0 knockdown and anti-PD-1 treatment resulted in significantly suppressed tumor growth and prolonged survival in mice, accompanied by elevated levels of IFN-γ and TNF-α in serum samples, indicating enhanced anti-tumor immunity.

**Conclusion:**

This study establishes a reliable risk model that complements TMB in guiding treatment decisions for ICI therapy. By incorporating the interaction between TMB and the TME, our model provides a more accurate prediction of patient prognosis and treatment response across multiple cancer types. The risk score's association with immunosuppressive TME components underscores the importance of considering the tumor's microenvironment in treatment planning. Furthermore, our findings highlight RPLP0 as a promising therapeutic target for combination immunotherapy. The robust predictive ability of our model across various cohorts and its potential to improve therapeutic outcomes offer new insights and directions for enhancing the efficacy of ICI therapy. Future research should focus on further validating this model in larger and more diverse cohorts, refining the gene set selection process, and exploring the specific mechanisms through which the identified biomarkers influence the TME and treatment response.

## 1 Introduction

Immune checkpoint inhibitor (ICI) therapy has become indispensable in the treatment of advanced tumors. As one of the most widely used ICI treatment options, PD1/PDL1 blockade significantly improves survival for a variety of tumors (1–4). However, only approximately 20% of patients benefit from ICI therapy for solid tumors (5). The low response rate of ICI treatment not only leads to over-treatment but also increases the incidence of adverse events (6). As the most widely studied biomarker, the correlation between tumor mutation burden (TMB) and ICI therapy response rate has been confirmed (7). In addition, TMB is associated with improved survival in ICI therapy across multiple cancer types (8). However, in practical applications, TMB still has several limitations. The prognostic value of TMB for overall survival (OS) has not been shown (9). The predictive ability of TMB for ICI treatment also differs for different tumor types (10). Here, we provide the perspective that the predictive ability of TMB in advanced tumor immunotherapy depends on its interaction with the tumor microenvironment (TME). The establishment of risk models to reflect the TME status based on data from the gene transcriptome is effective (11). We aimed to identify predictive biomarkers that can reflect the interaction between TMB and the TME to provide new ideas and targets for the precise individual treatment of ICI therapy.

## 2 Materials and methods

**Figure 1:**
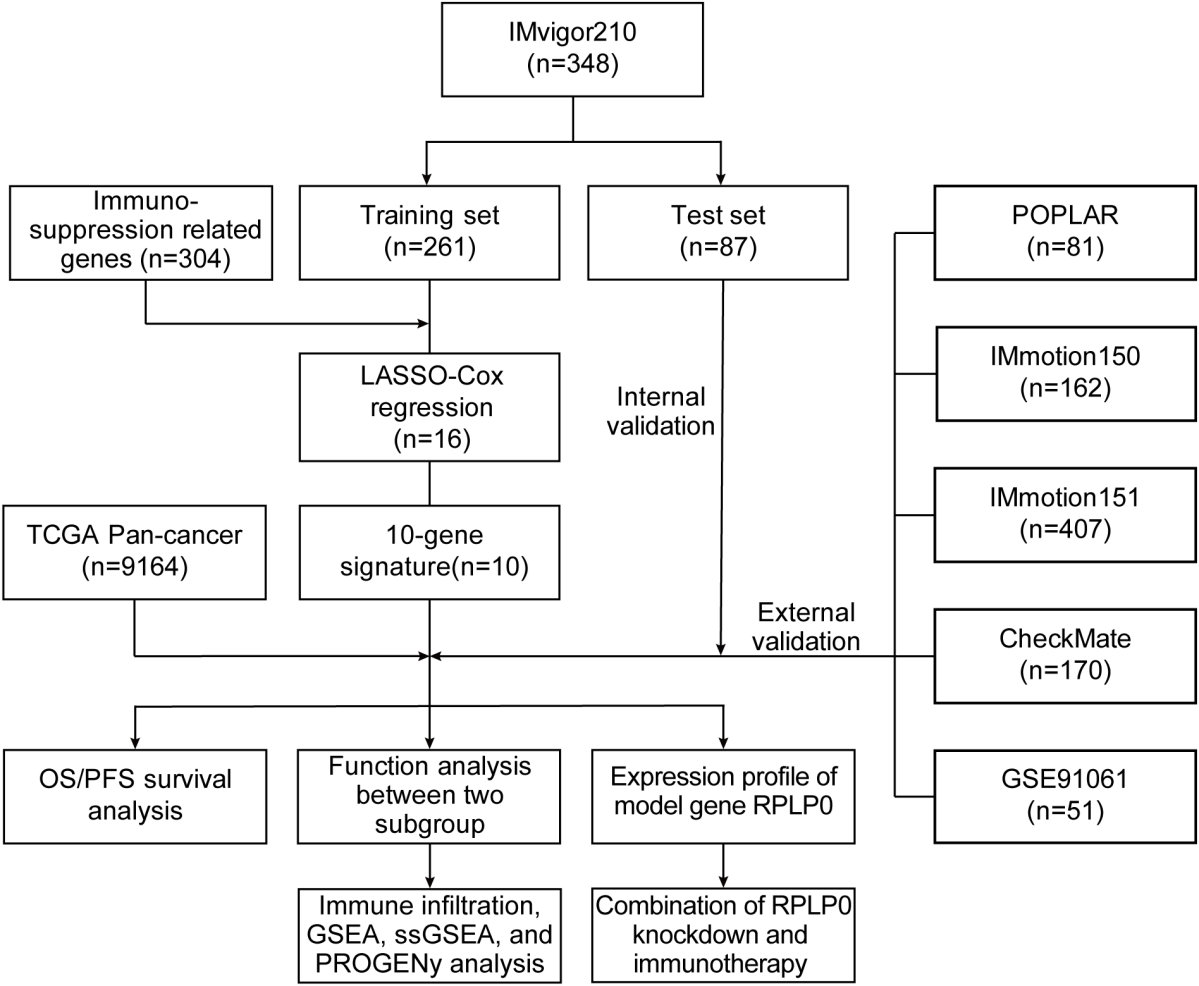
An overview of our methodology.

The flowchart of our methodology is exhibited in **Figure 1**.

### 2.1 Collection of genomic and clinical data

RNA-seq data before immunotherapy and matched clinical data of patients who received anti-PD1/PDL1 immunotherapy in several cohorts with different cancer types were collected. The expression profiles and corresponding clinical data of 348 patients with metastatic urothelial cancer (mUC) treated with atezolizumab (anti-PD-L1 antibody) were retrieved from the R package IMvigor210CoreBiologies (http://research-pub.gene.com/IMvigor210CoreBiologies) (4). Transcriptomic data of 208 patients with mUC, 162 patients with renal cell carcinoma (RCC), 81 patients with non-small cell lung cancer (NSCLC), and 206 patients with different cancer treated with the anti-PDL1 agent from four cohorts (IMvigor210, IMmotion150, POPLAR, and PCD4989g, respectively) were obtained from the European Genome-Phenome Archive [EGA, https://ega-archive.org/; study ID: EGAS00001004343 (12)]. Data from 407 patients treated with atezolizumab + bevacizumab from a clinical trial in RCC (IMmotion151) were collected from the EGA [study ID: EGAS00001004353 (13)]. Data from clinical trials of nivolumab (anti-PD1 antibody) were obtained from a report by Braun et al. (10) and Gene Expression Omnibus [GEO, https://www.ncbi.nlm.nih.gov/geo/, accession number: GSE91061 (14)]. These included 170 patients with advanced RCC from the CheckMate cohort and 51 patients with advanced melanoma from CA209-038, respectively. Patient clinical characteristics are given in **Supplementary Table S1**. RNA-seq data in transcripts per million (TPM) and the corresponding clinical data of TCGA Pan-Cancer were obtained from the UCSC Xena browser TCGA-hub (https://tcga.xenahubs.net). Patients with incomplete clinical information, gene expression data, and <30 days of OS or progression-free survival (PFS) were excluded.

### 2.2 Screening of immunosuppression-related genes

TMB represents the total number of somatic mutations per million bases in the exon coding region of a gene, including coding errors, base substitutions, base insertions, and base deletions (8). Theoretically, the higher the TMB of the tumor, the more neoantigens it produces, and the more likely it is to be recognized and killed by antitumor immune cells, meaning that immunotherapy might be more effective. However, in different solid tumors, the predictive ability of TMB for ICI treatment is different. Therefore, we hypothesized that the high expression of some genes, which interfered with the predictive role of TMB, weakened antitumor immunity. In our study, we conducted rigorous gene screening to identify 304 ISRGs from over 1,100 candidate genes. To address multiple hypothesis correction, we used the log-rank test and FDR method (FDR < 0.05) to balance rigorous correction and sensitivity. Our intersection approach retained genes significant in both IMvigor210 and CheckMate cohorts, ensuring robustness. This method also added validation by requiring consistent patterns across cohorts, reducing multiple testing issues and enhancing confidence in the biological relevance of the selected genes. After removing patients without TMB information in the IMvigor210 cohort, the median expression values of protein-coding genes were used to classify patients into high- and low-gene expression groups, respectively. Genes with *P* values of log-rank test <0.05 in the survival analysis were retained. Next, the patients were separated into high- and low-TMB groups according to the median. We further screened out a group of genes, whose high expression TMB group could not predict survival (log-rank *P >*0.05), while when the genes were in low expression, the patients in the high TMB group had better survival (log-rank *P <*0.05). After intersecting these genes with those whose *P* values were <0.05 in the survival analysis of OS in the CheckMate cohort, ISRGs (n = 304) were obtained.

### 2.3 Construction and assessment of the prognostic model

A total of 348 patients from the IMvigor210 cohort were randomly divided into training and internal validation (3:1 ratio) sets. Patients from the other cohorts were assigned to different external validation sets. Using the “glmnet” R package, we applied LASSO regression to pick and shrink the significant variables in the regression panel (15, 16). The OS data of the training set were the dependent variables in the regression, with an expression matrix of 304 ISRGs as the independent variables. The optimal lambda value was calculated using 1000-times cross-validation. Multivariable Cox regression analysis was conducted to establish a 10-gene-based risk prognostic model. The risk score for each patient was calculated using the formula below, where “exp” represents the expression level of genes and “coef” is the corresponding coefficient.


$$Risk\;Score=\sum_{i=1}^{n}\left(coef_{i}*\exp_{i}\right)$$

The patients were categorized into the high- and low-risk subgroups according to the median cut-off value of the training set or the optimal cut-off value calculated by the “survminer” R package. To assess the predictive ability of the model, the “pROC” R package was used to establish the receiver operating characteristic (ROC) curve and calculate the area under the curve (AUC).

### 2.4 Functional enrichment analysis and evaluation of TME components

We performed differential expression analysis between high- and low-risk subgroups using the “limma” R package. Genes ordered by log2(FoldChange) were inputted for Gene Set Enrichment Analysis (GSEA), and the results were visualized with the R package “clusterProfiler” (17, 18). Adjust P value <0.05 was considered statistically significant. The annotated gene set file of the hallmark gene sets (h.all.v7.5.1.symbols.gmt) was obtained from the Molecular Signatures Database (http://www.gsea-msigdb.org/). The CIBERSORT (19), ESTIMATE (20), and PROGENy (21) algorithms were used to measure the TME components and pathway activity. The “IOBR” R package was applied to calculate gene signature scores of hallmark and TME with single-sample gene set enrichment analysis (ssGSEA) algorithms (22). Running the “Limma” R package, significantly differential gene sets were identified by comparing the gene signature scores between the high- and low-risk groups (adjust P value < 0.05). The association between the risk score and ssGSEA scores was explored using Spearman correlation analysis.

### 2.5 RT-qPCR, western blot, and small interfering RNA (siRNA) interference

According to the manufacturer’s directions, we extracted the total RNA from tissues and cell lines using TRIzol Reagent. Then we conducted cDNA synthetization and quantitative real-time PCR (qRT-PCR) with a PrimeScript RT reagent kit and SYBR Green PCR reagent (EZBioscience, China). The primer sequences are given in **Supplementary Table S2**. With ACTB as an internal control, the mRNA relative expression levels were calculated using 2-ΔΔCT. The primary antibodies used in the western blot were as below: anti-alpha tubulin rabbit polyclonal antibody (11224-1-AP, Proteintech), anti-RPLP0 rabbit polyclonal antibody (11290-2-AP, Proteintech). The concentration was determined based on the manufacturer’s recommendation and practice. The western blot assays were conducted following the manufacturer’s protocol. We used genOFF™ *in vivo* siRNAs (RiboBio, China), specially designed to target *Rplp0*. These siRNAs undergo unique chemical modifications that bolster their serum stability without compromising efficiency. This enables direct intratumoral delivery, a straightforward and effective method requiring minimal dosage. The quick absorption of the siRNAs reduces experimental animal toxicity. In this experiment, cholesterol-modified *in vivo* siRNA targeting *Rplp0* for animal use was utilized. The siRNA sequences are provided in **Supplementary Table S3**.

### 2.6 *In vivo* mouse experiments

The mouse experiments were approved by the Institutional Ethics Committee for Clinical Research and Animal Trials Ethical of the First Affiliated Hospital of Sun Yat-sen University. 6~8 weeks old C57BL/6 mice were purchased and housed in specific-pathogen-free conditions. Mice were subcutaneously injected with MB49 cells (1 × 10^6^ cells/100 μL) and randomly divided into four groups (siCtrl, si*Rplp0*, siCtrl+αPD-1 and si*Rplp0*+αPD-1). One week after subcutaneous injection, si*Rplp0* or negative siCtrl were injected into tumors (5 nmol every 4 days, for a total of five times). For immunotherapy, mice were intraperitoneally injected with 100μg of anti-PD-1 antibody (BioXcell, USA) along with siRNA or siCtrl injection. We referenced published studies on gene knockdown using a similar intratumoral siRNA delivery method and our team’s previous work (23–25). Cholesterol-modified *in vivo* siRNA targeting *Rplp0* for animal use was injected into tumor-bearing mice to assess tumor size and growth. The volume of tumors was measured and calculated (volume = (length*width^2^)/2) every week. Such measurements were performed 4 times in total and stopped on day 28. Blood samples were collected on day 30 after subcutaneous injection. We obtained serum using serum separator tubes. The levels of IFN-γ and TNF-α in serum samples were determined through a mouse IFN-γ and TNF-α ELISA kit (Proteintch). We closely monitored and recorded the mice’s conditions daily. When mice reached humane endpoints—defined as tumor diameter exceeding 15mm, ≥15% weight loss, or distress signs like cyanosis and respiratory depression—we immediately euthanized them, dissected fresh tumors, snap-froze them in liquid nitrogen to preserve mRNA integrity, and stored them at -80°C. We also recorded survival times. Later, we analyzed tumor volumes and weights, and constructed survival curves.

### 2.7 Statistical analysis

Comparisons between high- and low-risk groups were conducted using the Wilcoxon rank-sum test. The distribution difference between the risk groups was tested using a two-sided Pearson’s chi-squared test. The correlation between two continuous variables was measured using Spearman correlation analysis. Kaplan–Meier (K–M) survival analysis with the log-rank test was used to compare survival between different subgroups. Univariate Cox regression (UniCox) analyses were conducted to assess the association between TMB, risk score, and survival in TCGA Pan-Cancer, which were visualized by the “forestplot” R package. All statistical analyses were performed using R software (version 4.1.0), and all statistical tests were two-tailed. Statistical significance was set at *P* < 0.05.

More details of materials and methods were given in **Supplementary Methods**.

## 3 Results

### 3.1 Influence of interactions between neoantigens and immune microenvironment on prognostic effect of TMB

**Figure 2:**
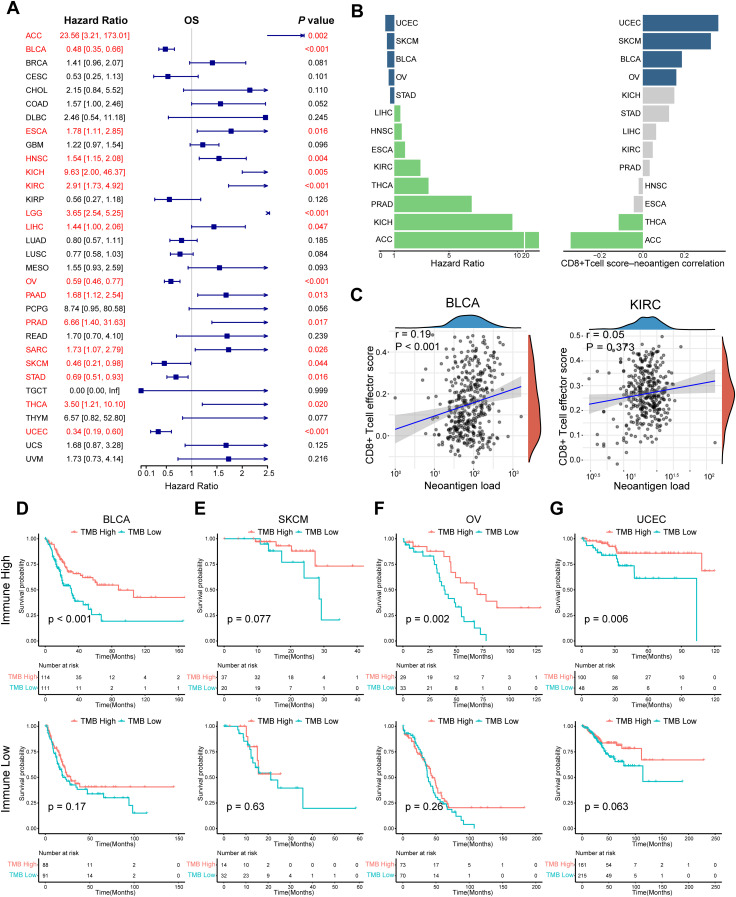
Impact of the interaction between neoantigens and the immune microenvironment on the prognostic effect of TMB. **(A)** Univariate Cox regression OS analysis of TMB in TCGA pan-cancer. Red color represents significant results (*P* < 0.05). **(B)** Differences in hazard ratio and correlation across multiple tumor types, in which TMB could predict prognosis. Grey color represents the *P* value of Spearman correlation was > 0.05. **(C)** Spearman correlation analysis between neoantigens and CD8+ T cell effector score in BLCA and KIRC. **(D–G)** K-M survival analysis of TMB in the high- and low-immune groups respectively in BLCA, SKCM, OV, and UCEC. Tumors were divided into high - and low - immune groups by unsupervised clustering. Hierarchical clustering was applied based on immune cell abundance, reflecting the differential immune cell infiltration in the tumor microenvironment. *P* values were calculated by log-rank test. OS, overall survival; TCGA, The Cancer Genome Atlas; TMB, tumor mutation burden. The abbreviation list of tumor cohorts from TCGA is given in **Supplementary Table S4**.

**Figure 3:**
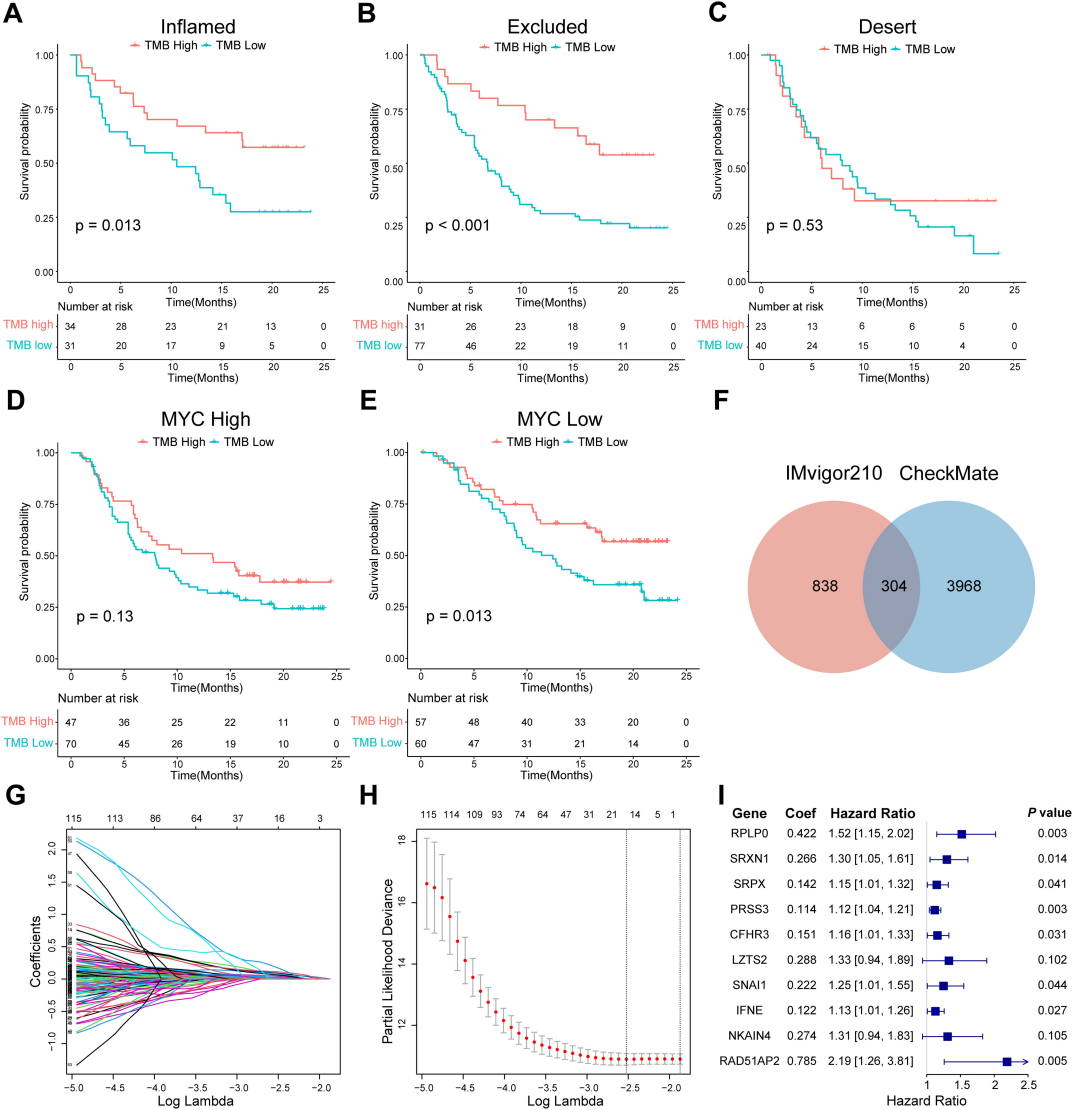
Screening of ISRGs and construction of the prognostic model in IMvigor210. **(A-C)** K-M survival analysis of TMB in three immunophenotypes in IMvigor210. P values were calculated by log-rank test. **(D, E)** K-M survival analysis of TMB in high- or low-MYC expression groups in IMvigor210, which was the example gene of the 1142 genes that remained. **(F)** Venn diagram showing the overlapping 304 genes. **(G-I)** LASSO regression profile showing gene coefficient changes across the regularization path, where increased regularization reduces minor gene coefficients toward zero, retaining only predictive genes. LASSO coefficient distribution map **(G)**, prognostic biomarker selection characteristics **(H)**, and forest plot based on multivariable Cox proportional hazards regression **(I)**. ISRGs, immunosuppression-related genes; K-M, Kaplan–Meier; TMB, tumor mutation burden.

TMB, one of the most extensively studied immunotherapy biomarkers, has been used to predict the efficacy of certain tumor immunotherapies. To further define its role in cancer, we analyzed 32 tumor datasets from TCGA (abbreviations in **Supplementary Table S4**). The effectiveness of TMB for survival prognosis was not consistent across different tumor types (**Figure 2A**). Given that the ability of TMB to generate neoantigens underlies its use as an immunotherapeutic biomarker, we analyzed the correlation between neoantigens and immune-infiltrating cells in different tumors. In tumors, such as bladder cancer and melanoma, TMB demonstrated good predictive power when neoantigen production was positively correlated with the degree of CD8+ cell infiltration (**Figures 2B, C**). Therefore, we propose that different immune microenvironments influence the predictive ability of TMB for prognosis, even within the same tumor. We divided bladder cancer, melanoma, and other tumors into immune-high and immune-low groups by unsupervised clustering and observed the effect of TMB in different subgroups (**Supplementary Figures S1A–D**). TMB demonstrated its most reliable predictive power in the hyper-immune group, which is characterized by high CD8+ T cell and M1 macrophage infiltration. However, this observation may not universally apply to all cancer types, such as OV and UCEC, where immune infiltration patterns differ. Further analysis is needed to clarify the predictive role of TMB across diverse tumor immunophenotypes (**Figures 2D–G**). Similarly, the differential predictive power of TMB was also observed in distinct immune phenotypes in the IMvigor210 cohort (**Figures 3A–C**). In conclusion, the predictive ability of TMB does not seem to represent its own function but depends on the interaction between the neoantigens it produces and the immune microenvironment. Therefore, the prediction characteristics of TMB can be different depending on changes in the microenvironment.

### 3.2 Construction of a prognostic model with IMvigor210 cohort

Next, we identified ISRGs in the ICI cohort. A total of 1142 genes were retained; when their expression was high, TMB could not accurately predict survival in the IMvigor210 cohort (**Figures 3D, E**). A total of 304 genes were maintained at the intersection of prognostic genes in the CheckMate cohort (**Figure 3F**, **Supplementary Table S5**). Through the LASSO regression analysis, 16 candidate ISRGs (RPS24, RPLP0, PLTP, SRXN1, SRPX, PRSS3, CFHR3, LZTS2, MATN3, BNC1, SNAI1, SERPINA5, IFNE, CCBE1, NKAIN4, and RAD51AP2) were selected (**Figures 3G, H**). Subsequently, a multivariable Cox regression analysis was applied to identify 10 optimal ISRGs (RPLP0, SRXN1, SRPX, PRSS3, CFHR3, LZTS2, SNAI1, IFNE, NKAIN4, and RAD51AP2) and construct a 10-gene prognostic model. The HR with 95% confidence intervals and coefficients of 10 genes are shown in the forest plots (**Figure 3I**).

### 3.3 Evaluation of the predictive efficiency of the prognostic model in the training and validation set

**Figure 4:**
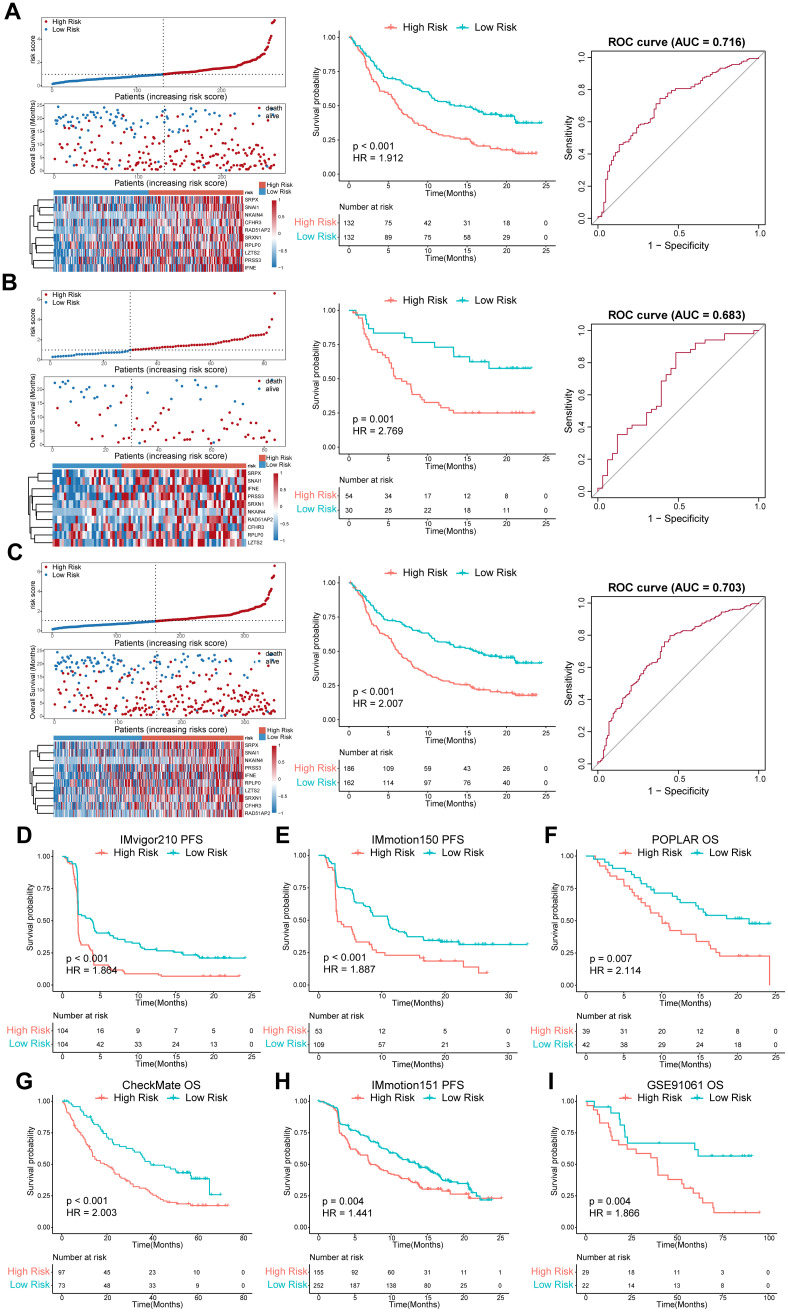
Evaluation of the predictive efficiency of the prognostic model in the training set, internal validation set, entire set, and external validation set. **(A-C)** left panel, distribution of the risk score, survival status along with survival times of patients, and heatmaps of the expression levels of the ten optimal ISRGs. The dotted line represents the median risk score of the training set and stratifies the patients into low- and high-risk groups; middle panel, K–M survival curves of OS; right panel, ROC curves of the predictive model for OS. **(D-I)** OS or PFS curves stratified by the low- and high-risk groups in different external validation sets across distinct tumor types. Data were analyzed by log-rank test. ISRGs, immunosuppression-related genes; K–M, Kaplan–Meier; OS, overall survival; PFS, progression-free survival; ROC, receiver operating characteristic curve.

**Figure 5:**
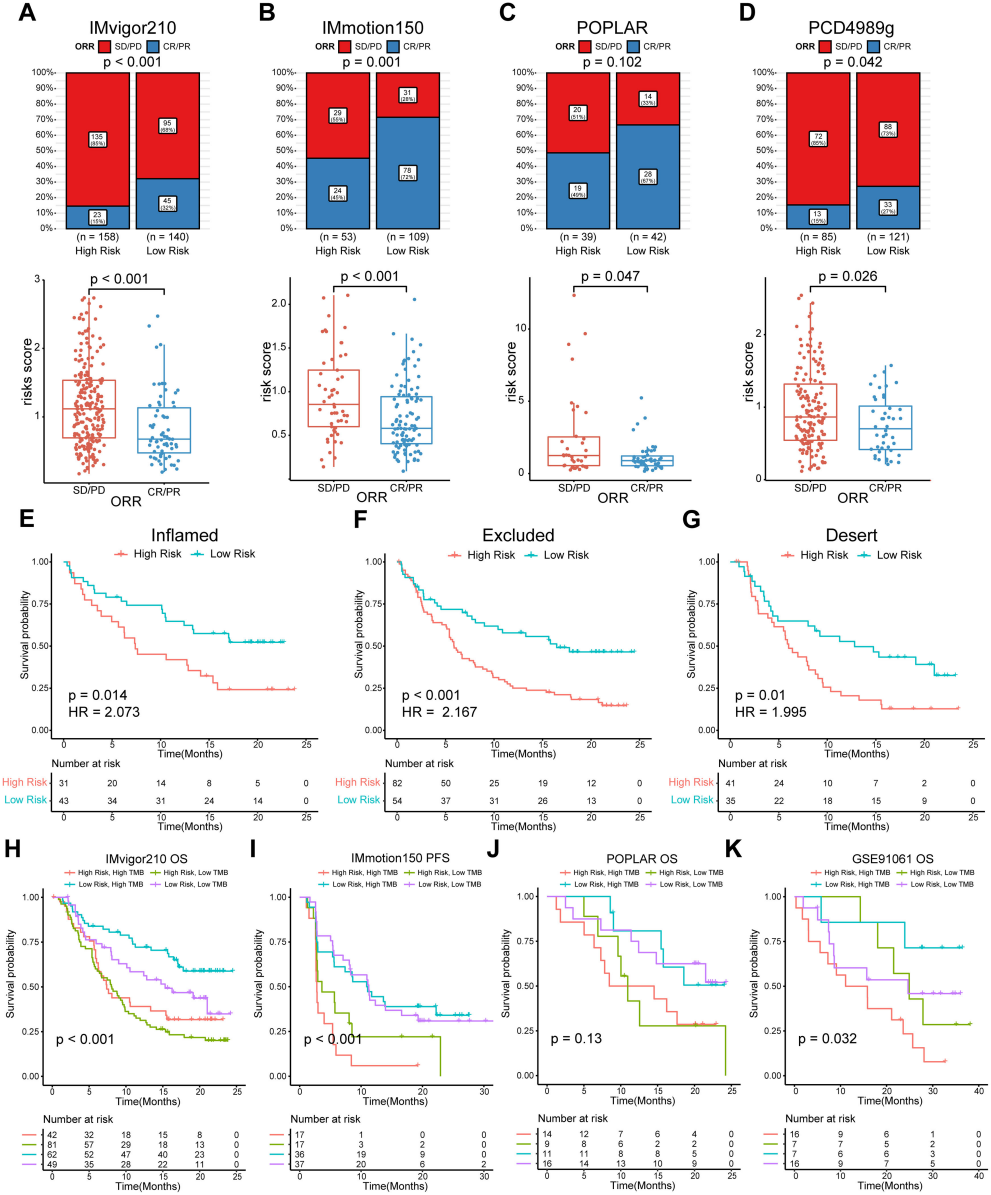
Evaluation of the predictive efficiency for ORR status of the model and subgroup analysis combined with TMB. **(A–D)** Top panel, bar charts representing the proportion of responders and non-responders by risk groups. Responders were defined as patients with complete (CR) or partial (PR) responses. Non-responders were defined as patients with stable (SD) or progressive (PD) disease. The distribution difference between the two groups was analyzed by two-sided Pearson’s chi-squared test; bottom panel, box plots representing the risk score by response group. Data were analyzed by Wilcoxon rank-sum test. **(E–G)** K-M survival analysis of the risk score in three immunophenotypes in IMvigor210. **(H–K)** K-M survival analysis of the risk score in TMB combinations in four indicated cohorts. ORR, objective response rate; K–M, Kaplan–Meier. TMB, tumor mutation burden.

We categorized patients into the high- or low-risk groups according to the median risk score of the training set (0.993) or the optimal cut-off value calculated by the “survminer” R package. The survival status of patients and the relative expression of the 10 genes in different risk groups in each set are shown in **Figures 4A–C**. Patients in the high-risk group had poorer OS than those in the low-risk group in the training set (HR = 1.912, *P* < 0.001), internal validation set (HR = 2.769, *P* = 0.001), and entire set (HR = 2.007, *P* < 0.001) (**Figures 4A–C**). Furthermore, patients with a higher risk score had shorter PFS (**Figure 4D**). ROC curves were applied to assess the predictive accuracy of the prognostic model, and the AUC of the training, internal validation, and entire set were 0.716, 0.683, and 0.703, respectively (**Figures 4A–C**). To further validate the predictive efficiency of the model in pan-cancer, we further analyzed the survival and ROC curves in the external validation set (**Figures 4E–I**, **Supplementary Figure S2**, **Supplementary Table S6**). In the atezolizumab arm of four cohorts among different tumor types, the low-risk group exhibited an increased proportion of responders (**Figures 5A–D**). Significant differences in risk score levels were observed between responders and non-responders, indicating the predictive value of this model for response to atezolizumab (**Figures 5A–D**). Based on CD8^+^T cell infiltration, urothelial cancer was divided into three immune subtypes: inflamed, excluded, and desert. Patients with higher risk scores had poorer OS despite the immune subtype (**Figures 5E–G**), suggesting a superior predictive value of our model compared with TMB. To verify that the predictive ability of TMB depends on the immune suppression factors in the TME, we divided patients into four subgroups according to TMB and risk score across multiple cancer types. The survival analysis showed that low-score high-TMB patients had the best prognosis, while high-score high-TMB patients had the poorest survival, which was consistent with our hypothesis (**Figures 5H–K**). In brief, our model showed an excellent predictive capacity for survival and response in several immunotherapy cohorts across multiple cancer types.

### 3.4 Analysis of correlation between risk score and TME components

The CIBERSORT and ESTIMATE algorithms were applied to access the TME components. In the IMvigor210 cohort, the abundances of Tregs, M0 macrophages, and activated mast cells in the high-risk group were significantly higher than those in the low-risk group, while the abundances of gamma delta T cells, CD4 naïve cells, T cells follicular helper cells, and M1 macrophages had the opposite trend (**Supplementary Figure S3A**). Furthermore, Spearman correlation analysis revealed that the risk score was negatively correlated with the abundance of antitumor immune cells, such as CD8 T cells, gamma delta T cells, CD4 memory-activated T cells, follicular helper T cells, and M1 macrophages. The risk score was positively correlated with the abundance of M0 macrophages and activated mast cells (**Supplementary Figures S3D–J**). The IMmotion150 and POPLAR data sets showed a trend of immune infiltration that was relatively consistent with the IMvigor210 cohort (**Supplementary Figures S3B, C, K–M**). The results of ESTIMATE showed that the stromal score was conspicuously higher in the high-risk group in the IMvigor210 (P < 0.001, **Supplementary Figure S3N**) and IMmotion150 cohorts (P < 0.01, **Supplementary Figure S3O**), while the immune score was higher in the low-risk group in the POPLAR cohort (P < 0.01, **Supplementary Figure S3P**). Taken together, the high-risk group showed a more suppressive TME status than the low-risk group, across all three cohorts.

### 3.5 Functional enrichment analysis, ssGSEA, and PROGENy pathway activity assessment

To investigate the underlying mechanism relevant to the ISRG risk model contributing to the immunosuppressive status in the TME, we conducted functional enrichment analysis. The results of GSEA analysis based on hallmark indicated that epithelial–mesenchymal transition (EMT), hypoxia, glycolysis, E2F targets, MYC targets, and the G2M checkpoint pathway were enriched in high-risk patients across multiple cancer types, whereas immune responses, such as interferon response, were negatively correlated with high-scoring risks in RCC, NSCLC, and SKCM (**Supplementary Figures S4A–D**). Intriguingly, inflammatory responses were activated in high-risk patients with mUC. We further assessed the hallmark and TME gene set scores using the ssGSEA algorithms. The heatmap of the IMvigor210 cohort suggested that a high-risk score was closely associated with EMT, hypoxia, myeloid-derived suppressor cells (MDSC), and gene signatures featuring stromal components, including cancer associated fibroblast (CAF) and TGF-β family members (**Supplementary Figure S5A**), which was similar with the above results. TME signature scores related to the activation of antitumor immune cells, such as CD8 T and cytotoxic cells, were elevated in the low-risk group, while hypoxia and signatures related to immune suppression, such as MDSC and Th2 cells, were positively correlated with high-scoring risks in the IMmotion150 and POPLAR cohorts (**Supplementary Figure S5C, E**). The difference in hallmark signature scores between the high- and low-risk groups was roughly consistent with the GSEA results (**Supplementary Figures S5B, D, F**). Activity scores of signaling pathways were calculated using PROGENy algorithms. The high-risk group in the IMvigor210 cohort had a significantly higher score than the low-risk group for most malignant signaling pathways, including TGF-β, EFGR, hypoxia, Wnt, and TNFα (**Supplementary Figure S6A**). Similar results and tendencies were observed in the IMmotion150 and POPLAR cohorts (**Supplementary Figures S6B, C**). Overall, these comparative analyses revealed that hyperactivity of stromal components, EMT, and hypoxia in high-risk subsets might contribute to the poor survival of immunotherapy patients and lay the foundation for further dissecting the potential molecular mechanisms.

### 3.6 Prognostic value and exploration of the mechanism of the model in TCGA pan-cancer

**Figure 6:**
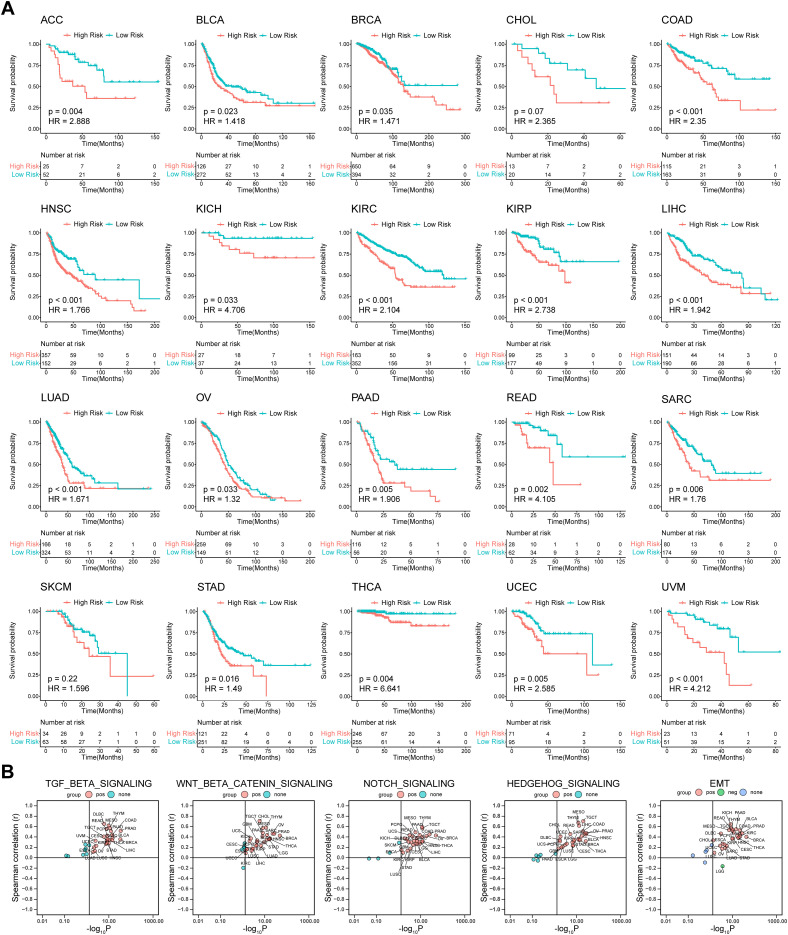
Prognostic value of the predictive model and correlation analysis between the risk score and ssGSEA score in TCGA Pan-Cancer. **(A)** K-M OS analysis of the risk score in TCGA pan-cancer in the indicated tumor types. *P* values were calculated by log-rank test. **(B)** Spearman correlation analysis between the risk score and ssGSEA score of hallmark. ssGSEA, single-sample gene set enrichment analysis; K-M, Kaplan–Meier; OS, overall survival. The abbreviation list of 32 tumor cohorts from TCGA is given in **Supplementary Table S4**.

To verify the predictive ability of our model for prognosis in TCGA pan-cancer, survival analysis, and UniCox were performed for the 32 cancer types. The Kaplan-Meier OS analysis showed that an elevated risk score was associated with inferior prognosis across multiple cancers, such as ACC, BLCA, COAD, HNSC, KICH, KIRC, KIRP, LIHC, LUAD, PAAD, READ, SARC, STAD, THCA, UCEC, and UVM (**Figure 6A**). UniCox results indicated that a higher risk score predicted worse OS and disease-specific survival (DSS) status of patients for most cancer types (**Supplementary Figure S7**). Collectively, these results suggest that the prognostic capacity of our model was satisfactory and revealed its potential as a biomarker for multiple cancers. Given the significant association between the risk subgroups and stromal and immunosuppressive components in the TME, Spearman correlation analysis between the risk score and ssGSEA score of hallmark and TME was utilized to further explore whether this association can be extended to pan-cancer. The results of hallmark showed that the risk score was strongly positively correlated with TGF beta, Wnt/β-catenin, NOTCH, and HEDGEHOG signaling (**Figure 6B**), which are pro-tumor signaling pathways, across pan-cancer. Consistent with the above results, the risk score displayed a strong positive correlation with EMT, glycolysis, angiogenesis, and hypoxia signatures (**Figure 6B**, **Supplementary Figure S8A**), whereas it was anti-correlated with oxidative phosphorylation in various cancers. For the TME signature, the risk score was positively correlated with MDSC and CAF and negatively correlated with T cells and cytotoxic cells (**Supplementary Figure S8B**). Collectively, these results shed light on the possible cellular biological mechanisms of ISRGs across pan-cancer.

### 3.7 Therapeutic potential of RPLP0 knockdown and anti-PD-1 combination therapy in BLCA

**Figure 7:**
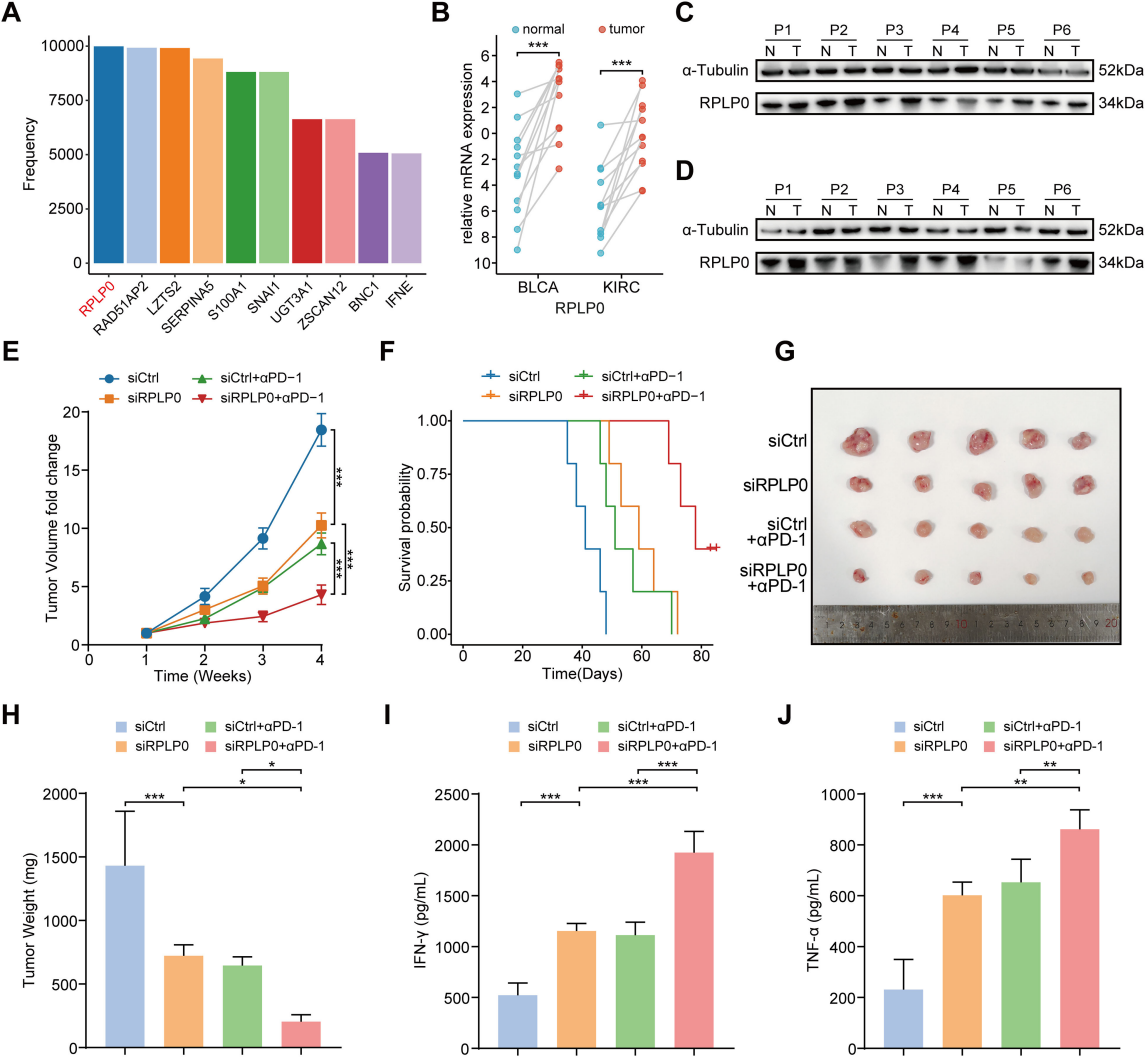
Therapeutic potential of RPLP0 knockdown and anti-PD-1 combination therapy in BLCA subcutaneous tumor model. **(A)** Bar charts representing the frequency of remaining optimal genes after LASSO regression with 10000 cycles training in IMvigor210. **(B)** mRNA expression level of RPLP0 in BLCA and KIRC patients by qRT-PCR (n = 12). P value was determined by paired t-test. **(C, D)** Protein expression of RPLP0 detected by Western blot in paired normal tissues and BLCA **(C)**, KIRC tissues **(D)**. **(E–H)** Tumor growth curves **(E)**, survival curves **(F)**, tumor images **(G)**, and tumor weight **(H)** of mice under the indicated treatments (n = 5 per group). **(I, J)** Bar charts representing the levels of IFN-γ **(I)** and TNF-α **(J)** detected by ELISA in serum samples from mice in different groups. One-way ANOVA followed by Tukey’s multiple comparisons tests were used to compare data in four groups. BLCA, bladder urothelial carcinoma; ELISA, enzyme-linked immunosorbent assay; KIRC, kidney renal clear cell carcinoma. P < 0.05, ** P < 0.01, ***P < 0.001.

To identify robust biomarker genes in the model, we conducted high-frequency LASSO screening on the IMvigor210 dataset and recorded the number of times each gene was selected. We observed that RPLP0 ranked extremely high in the number of times it was selected, even in the case of mixed tumor factors (**Figure 7A**), which suggests that it is a robust biomarker. Since RPLP0 showed prominent expression in these cancers in pan-cancer analysis, and was frequently selected in IMvigor210 cohort screening. Data from specific databases like IMvigor210 facilitated research. We aimed to explore cancers with poor immune-therapy responses, and BLCA and KIRC showed lower response rates. Thus, our subsequent research will predominantly concentrate on BLCA and KIRC. Then we compared the expression level of RPLP0 in adjacent normal tissue and tumors from clinical samples of KIRC and BLCA. The qRT-PCR and western blot results indicated that RPLP0 was significantly overexpressed in tumors (**Figures 7B–D**). Concordantly, the RPLP0 protein expression profile in pan-cancer and immunohistochemistry images from the Human Protein Atlas database also demonstrated the elevated protein expression of RPLP0 in tumor tissues (**Supplementary Figures S9A, B**). To explore the synergistic effect of targeting *Rplp0* in immunotherapy, we established the subcutaneous BLCA model (MB49 cells in C57BL/6 mice) and administered different treatments in four groups. Treatment of cholesterol-modified *in vivo* siRNAs specifically targeting *Rplp0* in tumors significantly reduced *Rplp0* expression in tumors (**Supplementary Figure S10**). The result indicated that the combination of *Rplp0* knockdown and anti-PD-1 treatment prominently suppressed tumor growth and prolonged survival of mice compared to si*Rplp0* or immunotherapy monotherapy (**Figures 7E–H**). Through enzyme-linked immunosorbent assay (ELISA), we found *Rplp0* knockdown and immunotherapy combination remarkably augmented the IFN-γ and TNF-α levels in serum (**Figures 7I, J**), which implies combinational treatment potentiated the anti-tumor immunity in mice. These results confirmed the abnormal expression of RPLP0 in tumors and its potential role as a therapeutic target in immunotherapy.

## 4 Discussion

ICI therapy has broad prospects in the treatment of many tumors. However, neither clinical pathology nor genomics research has found any biological prediction index consistent with ICI treatment response. Among the existing biomarkers, TMB is considered a unified variable for predicting the therapeutic effect of ICIs in pan-cancer. However, TMB has many limitations in clinical applications. First, the predictive ability of TMB is mainly reflected in the ICI treatment response and disease remission rates, and its predictive or prognostic value for OS has not been confirmed (9). Second, there are huge differences in TMB data between different tumor types and individuals (26). For example, although there is only a moderate amount of TMB in renal cell carcinoma, the effect of ICI treatment on renal cell carcinoma is not poor. Additionally, TMB in renal cell carcinoma is not related to the therapeutic effects of ICIs (10, 26). There is no consensus on the determination of the critical value of TMB risk stratification in ICI treatment; high-TMB failed to predict ICI response across all cancer types (27).

The accumulation of somatic mutations in tissues is an important driving factor in tumor transformation. TMB is defined as the accumulation of nonsynonymous somatic mutations in the coding region (7). Interestingly, TMB was higher in tumors with a higher pathological grade. This suggests that TMB is closely related to the degree of malignancy of the tumor, contrary to the expectation. Somatic mutations can be expressed at the RNA or protein level by transcription or expression. The greater the number of non-synonymous mutations in tumors, the higher the possibility of producing new antigens and the more likely it is to activate T cells and cause an immune response. However, not all newly produced polypeptides or proteins have immunogenicity (28). TMB cannot completely represent new immunogenic antigens. Notably, many factors, such as the infiltration level and activity of T cells in tumors, tumor metabolism, and immune checkpoint expression, will affect the recognition and response of the TME to new antigens (29). High TMB exhibited heterogeneous immune infiltration: in certain cancers, it associated with pronounced CD8+ T cell and M1 macrophage infiltration, indicative of an active yet suppressed immune response; conversely, low infiltration in others implied immune evasion or a poorly immunogenic milieu. These results highlight the intricate TMB-immune infiltration relationship. Given the variability of immune infiltration across cancer types, the role of TMB in immune prognosis should be generalized cautiously. Overall, this analysis enhances the understanding of TMB and immune infiltration’s interplay in cancer prognosis as presented in **Figure 2A**. In summary, judging from the complexity of the immune response and the characteristics of TMB itself, TMB has limitations as a predictive biomarker, especially when used alone. Therefore, we propose that the predictive ability of TMB is based on the interaction between new antigens and the immune microenvironment. Even in different microenvironments of the same tumor, the predictive ability of TMB will have completely different results. For example, in mUC, high-TMB tumors responded well to ICI treatment. After subdividing mUC into sub-categories according to the immune microenvironment, TMB retained its predictive ability in the high immune group, but TMB in the low immune group could not separate the clinical outcomes of patients, whether they received ICI treatment or not.

It is feasible to establish risk characteristics by transcriptome analysis to monitor the immune status of tumors and guide individualized immunotherapy (11, 30). Therefore, based on this interesting characteristic of TMB, we screened some target genes by analyzing the transcriptome data of the ICI treatment cohort of mUC. For example, MYC is activated by genetic, epigenetic, or post-translational mechanisms in most cancers, and it can inhibit the immune response against these cancers in various ways (31, 32). We then used the LASSO-Cox method to establish the related risk model and verified it using the ICI treatment dataset of multiple cancers. In the independent ICI treatment datasets of multiple tumors, our model showed good prognosis prediction ability. We observed a similar situation in TCGA Pan-cancer. This suggests that the gene set screened using this method may have a relatively consistent role in pan-cancer. Furthermore, functional enrichment analysis, ssGSEA, and correlation analysis between the risk score and ssGSEA score corroborated that tumors with higher risk scores had higher activation of TGF-β signaling, hypoxia, EMT, angiogenesis, and stromal components, such as ECM, CAF, and MDSC, across pan-cancer. The TGF-β signaling pathway, which attenuates the function of adaptive and innate immune cells and is linked with tumor immune evasion (33), can shape the heterogeneity of CAF (34) and induce the polarization of MDSC (35), resulting in the suppressive status of the TME. Due to the important role of the TGF-β pathway in the TME, various small-molecule compounds targeting TGF-β signaling are currently in preclinical experiments (36). Considering the obvious differences in TGF-β signaling between high- and low-risk groups, combined immunotherapy, anti-TGF-β therapy, and anti-angiogenesis therapy for patients in high-risk groups may be a future research direction with great potential.

In the process of variable selection and model building, we observed an interesting phenomenon. When a model was determined to be suitable for pan-cancer, there was always a ribosomal protein RPLP0, which ranked high in the number of times being selected in high-frequency LASSO screening in IMvigor210 cohorts. Besides forming ribosomes and participating in protein biosynthesis, ribosomal proteins are closely related to cancer initiation and progression (37–39). It has been reported that RPLP0, a member of the ribosomal P complex family, was upregulated in gynecologic tumors and involved in the process of tumor development (40–42). Herein, we confirmed the overexpression of RPLP0 in BLCA and KIRC tissues with clinical samples. Through *in vivo* mouse experiments, siRNA interference, and ELISA, we demonstrated that RPLP0 knockdown in tumors and anti-PD-1 combinational treatment remarkably inhibited tumor growth and improved anti-tumor immunity.

Although our research shows good predictive value in multiple tumors, it is mainly focused on a retrospective study of existing data, its applicability is limited, and it still needs to be further verified in larger cohorts and in other populations. Different tumors can share an optimal cut-off value, but ideally, the optimal cut-off value should be chosen based on the conditions of each tumor. The risk model, initially developed using a bladder cancer cohort (IMvigor210), demonstrated robust predictive performance across diverse tumor types and treatment settings (including monotherapy and combination therapy), suggesting its potential cross-tumor and cross-treatment applicability. However, employing distinct optimal risk cut-offs (e.g., the median risk score from the training set or dynamic thresholds derived via the “survminer” R package) in validation cohorts may introduce optimistic bias in model evaluation, as these adjustments aim to align with cohort-specific characteristics to enhance real-world validity. Future studies should adopt the following strategies to harmonize evaluation consistency: 1. Fixed cut-off: Apply the median risk score from the training cohort as a universal threshold to ensure standardized criteria, though its generalizability requires validation. 2. Dynamic cut-off: Optimize thresholds based on cohort-specific characteristics, necessitating statistical adjustments to mitigate overfitting risks. 3. Multi-method integration: Combine multiple thresholding approaches with sensitivity analyses to strengthen robustness. Strategy selection should weigh study objectives (e.g., clinical rigor vs. exploratory research) and cohort heterogeneity, with transparent reporting of methodological limitations. In the future, we can attempt to screen the gene set in a more elaborate manner and build a better model.

Although we selected RPLP0 as a biomarker candidate, its roles in TME and the specific mechanism should be further investigated *in vitro* and *in vivo*. We found that RPLP0 is highly expressed in tumor tissues and RPLP0 may regulate immune cell infiltration and activity in the TME. For instance, RPLP0 might affect the recruitment or function of immunosuppressive cells like MDSCs or Tregs, which are known to suppress anti-tumor immune responses. Moreover, RPLP0’s role in immune regulation may stem from its function in ribosomal biogenesis and protein synthesis. As a ribosomal protein, RPLP0 participates in mRNA translation. Its dysregulation may lead to the production of specific proteins that alter the TME. These proteins could include cytokines or chemokines affecting immune cell migration, as well as factors promoting angiogenesis or extracellular matrix remodeling, all of which may influence immune cell entry into tumors. However, the exact pathways and mechanisms underlying these effects of RPLP0 remain to be clarified. To address these knowledge gaps, we propose the following research directions: 1. *In vitro* mechanistic studies: Perform co-culture experiments of tumor cells with immune cells to determine the impact of RPLP0 on immune cell function. Also, conduct transcriptomic and proteomic analyses of RPLP0-knocked-down tumor cells to identify downstream effector molecules and pathways. 2. Analyzing RPLP0’s role in immune cell infiltration: Apply advanced technologies like flow cytometry and single-cell RNA sequencing to analyze the immune cell landscape in RPLP0-knocked-down tumors. 3.Exploring RPLP0-tumor stroma interactions: Study how RPLP0 interacts with stromal components such as cancer-associated fibroblasts and the extracellular matrix. Investigate the paracrine signaling pathways that RPLP0 may be involved in. 4. Clinical correlation studies: Analyze clinical datasets to correlate RPLP0 expression with immune infiltration patterns and patient outcomes. Although RPLP0 shows promise as a biomarker and therapeutic target, a more in-depth investigation of its mechanisms in influencing the TME and immune responses is essential. This will facilitate the development of more effective immunotherapeutic strategies.

In summary, our classifier seems to be a useful and reliable prediction tool, which can provide the prognostic value of ICI treatment for pan-cancer and can supplement the existing prediction systems to achieve more accurate individual treatment. Moreover, RPLP0 may be a valuable biomarker and therapeutic target for patients treated with ICIs.

## Data Availability

The data presented in the study are deposited in the European Genome-Phenome Archive (EGA) repository, accession number EGAS00001004343 and EGAS00001004353, and the Gene Expression Omnibus (GEO) repository, accession number GSE91061. Additionally, the data sets can be downloaded from the IMvigor210CoreBiologies R package repository, data URL: http://research-pub.gene.com/IMvigor210CoreBiologies, the UCSC Xena TCGA Hub repository, data URL: https://tcga.xenahubs.net, the UALCAN portal repository, data URL: http://ualcan.path.uab.edu, and/or the Human Protein Atlas repository, data URL: https://www.proteinatlas.org.

## Glossary

AUC: Area under the ROC curve
CAF: Cancer associated fibroblast
DSS: Disease-specific survival
ECM: extracellular matrix
EMT: Epithelial–mesenchymal transition
ELISA: enzyme-linked immunosorbent assay
GSEA: Gene Set Enrichment Analysis
ICI: Immune checkpoint inhibitor
ISRGs: Immunosuppression-related genes
K–M: Kaplan–Meier
MDSC: Myeloid-derived suppressor cells
mUC: Metastatic urothelial cancer
NSCLC: Non-small cell lung cancer
ORR: Objective response rate
OS: Overall survival
PFS: Progression-free survival
qRT-PCR: quantitative real-time PCR
ROC: Receiver operating characteristic curve
RCC: Renal cell carcinoma
siRNA: small interfering RNA
ssGSEA: Single-sample gene set enrichment analysis
TCGA: The Cancer Genome Atlas
TMB: Tumor mutation burden
TME: Tumor microenvironment
TPM: Transcripts per million
UniCox: Univariate Cox regression.
