# Supplementary material for: Interplay between tumor mutation burden and the tumor microenvironment predicts the prognosis of pan-cancer anti-PD-1/PD-L1 therapy
Source: Front Immunol. 2025 Jul 24;16:1557461. doi: 10.3389/fimmu.2025.1557461 (PMC12328289; doi:10.3389/fimmu.2025.1557461)
Supplement: Supplementary file 1 [file DataSheet1.docx]

**Supplementary Figure**

**Figure S1:** Heatmap of 22 immune cell types based on immune microenvironment clustering.

**Figure S2:** Evaluation of the predictive efficiency of the prognostic model.

**Figure S3:** Difference of TME components between high- and low-risk groups.

**Figure S4:** GSEA analysis based the genelist ordered by log2(FoldChange).

**Figure S5:** Heatmap of ssGSEA scores of TME and hallmark gene sets.

**Figure S6:** Difference of pathways activity between the high- and low-risk groups.

**Figure S7:** Univariate Cox regression analysis of risk score.

**Figure S8:** Correlation analysis between risk score and ssGSEA score in in different cancer types from the TCGA dataset.

**Figure S9:** RPLP0 protein expression profile in normal tissues and tumors.

**Figure S10**: RPLP0 mRNA expression level in subcutaneous tumors.

**Supplementary Table**

**Table S1:** Patient clinical characteristics.

**Table S2:** Oligo sequences used in quantitative real-time PCR.

**Table S3:** siRNA target sequence used for gene knockdown.

**Table S4:** Abbreviation of 32 TCGA cancer types.

**Table S5:** Details of 304 immunosuppression-related genes (ISRGs).

**Table S6:** Univariate Cox regression of commonly used characteristics and risk score in immune checkpoint inhibitor (ICI) treatment sets.

**Supplementary method**

Genomic and clinical data sources and preprocessing.

Clinical samples collection.

Cell line.
