# Supplementary material for: Interplay between tumor mutation burden and the tumor microenvironment predicts the prognosis of pan-cancer anti-PD-1/PD-L1 therapy
Source: Front Immunol. 2025 Jul 24;16:1557461. doi: 10.3389/fimmu.2025.1557461 (PMC12328289; doi:10.3389/fimmu.2025.1557461)
Supplement: Supplementary file 2 [file DataSheet2.pdf]

Supplementary Figure

Figure S1: Heatmap of 22 immune cell types based on immune microenvironment clustering.

Figure S2: Evaluation of the predictive efficiency of the prognostic model.

Figure S3: Difference of TME components between high- and low-risk groups.

Figure S4: GSEA analysis based the genelist ordered by log2(FoldChange).

Figure S5: Heatmap of ssGSEA scores of TME and hallmark gene sets.

Figure S6: Difference of pathways activity between the high- and low-risk groups.

Figure S7: Univariate Cox regression analysis of risk score.

Figure S8: Correlation analysis between risk score and ssGSEA score in in different cancer types from the TCGA dataset.

Figure S9: RPLP0 protein expression profile in normal tissues and tumors.

Figure S10: RPLP0 mRNA expression level in subcutaneous tumors.

Supplementary Table

Table S1: Patient clinical characteristics.

Table S2: Oligo sequences used in quantitative real-time PCR.

Table S3: siRNA target sequence used for gene knockdown.

Table S4: Abbreviation of 32 TCGA cancer types.

Table S5: Details of 304 immunosuppression-related genes (ISRGs).

Table S6: Univariate Cox regression of commonly used characteristics and risk score in immune checkpoint inhibitor (ICI) treatment sets.

Supplementary method

Genomic and clinical data sources and preprocessing.

Clinical samples collection.

Cell line.

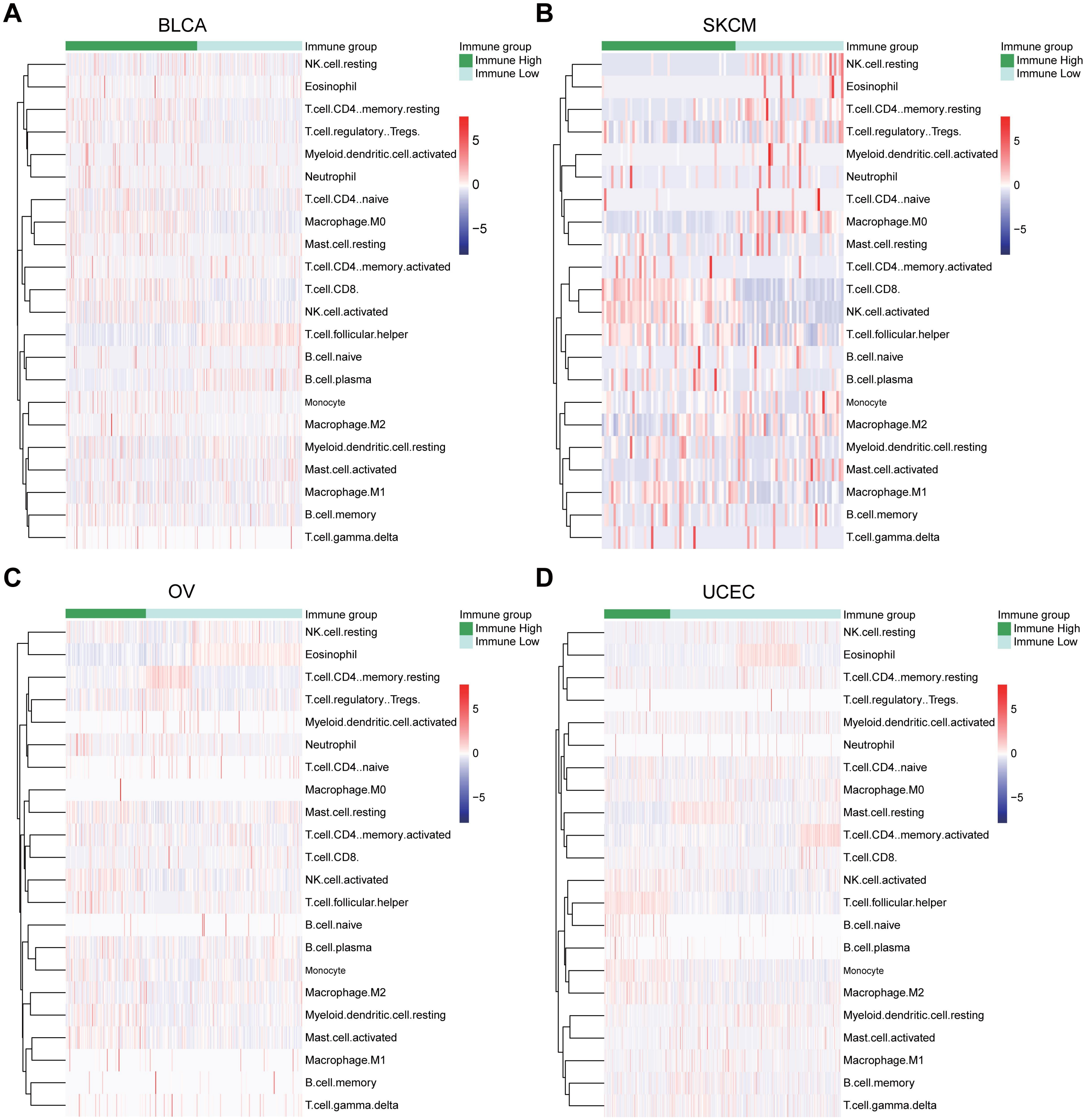

Figure S1. Heatmap of 22 immune cell types based on immune microenvironment clustering. **(A-D)** Heatmap of 22 immune cell types based on consensus clustering identifying the high- and low-immune subgroups in BLCA, SKCM, OV and UCEC. Abbreviation list of tumor cohorts from TCGA is given in **Table S4**.

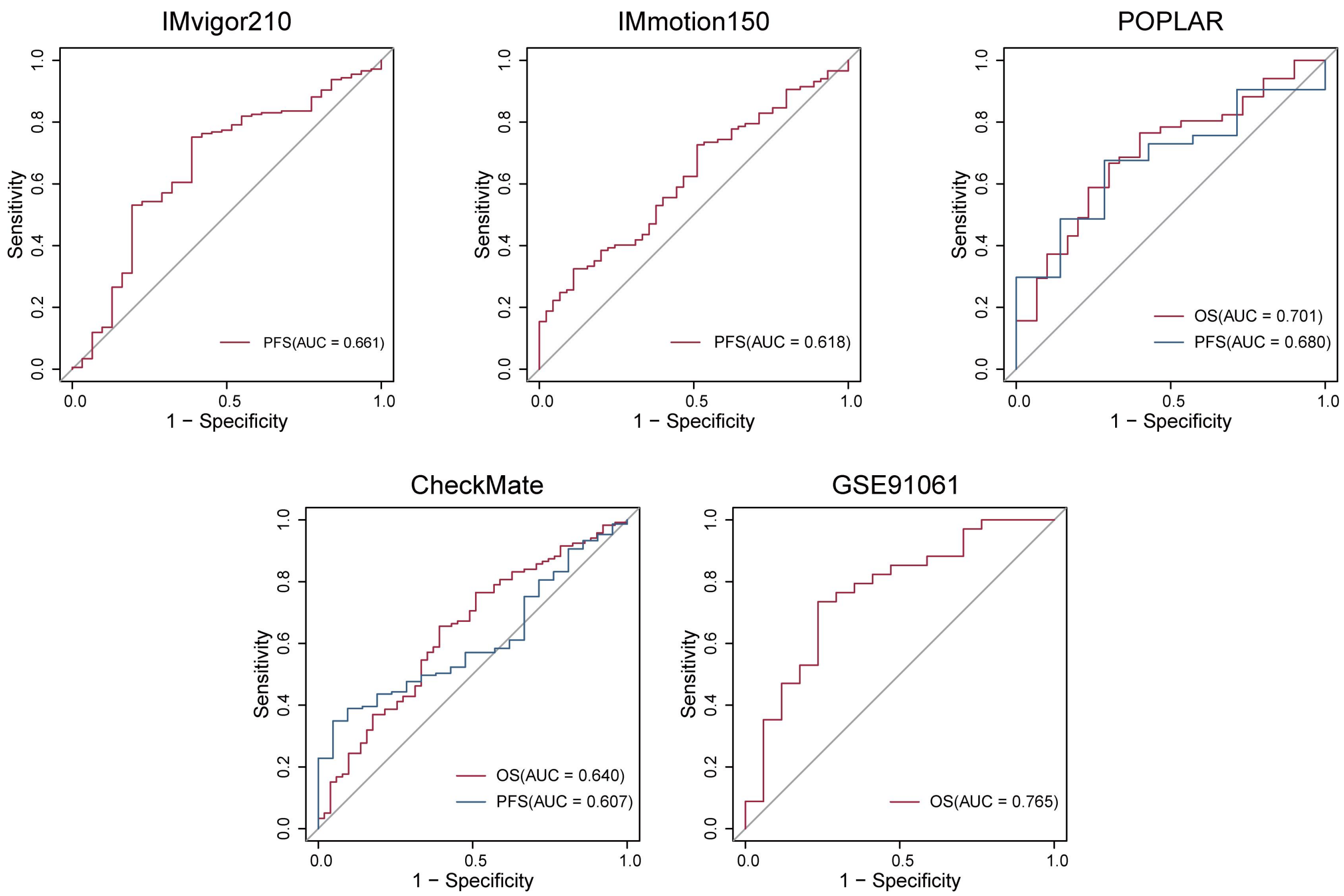

Figure S2. Evaluation of the predictive efficiency of the prognostic model. ROC curves representing the predictive capacity for OS and PFS of prognostic model in the indicated immunotherapy cohorts. OS, overall survival; PFS, progression-free survival; ROC, receiver operating characteristic curve.

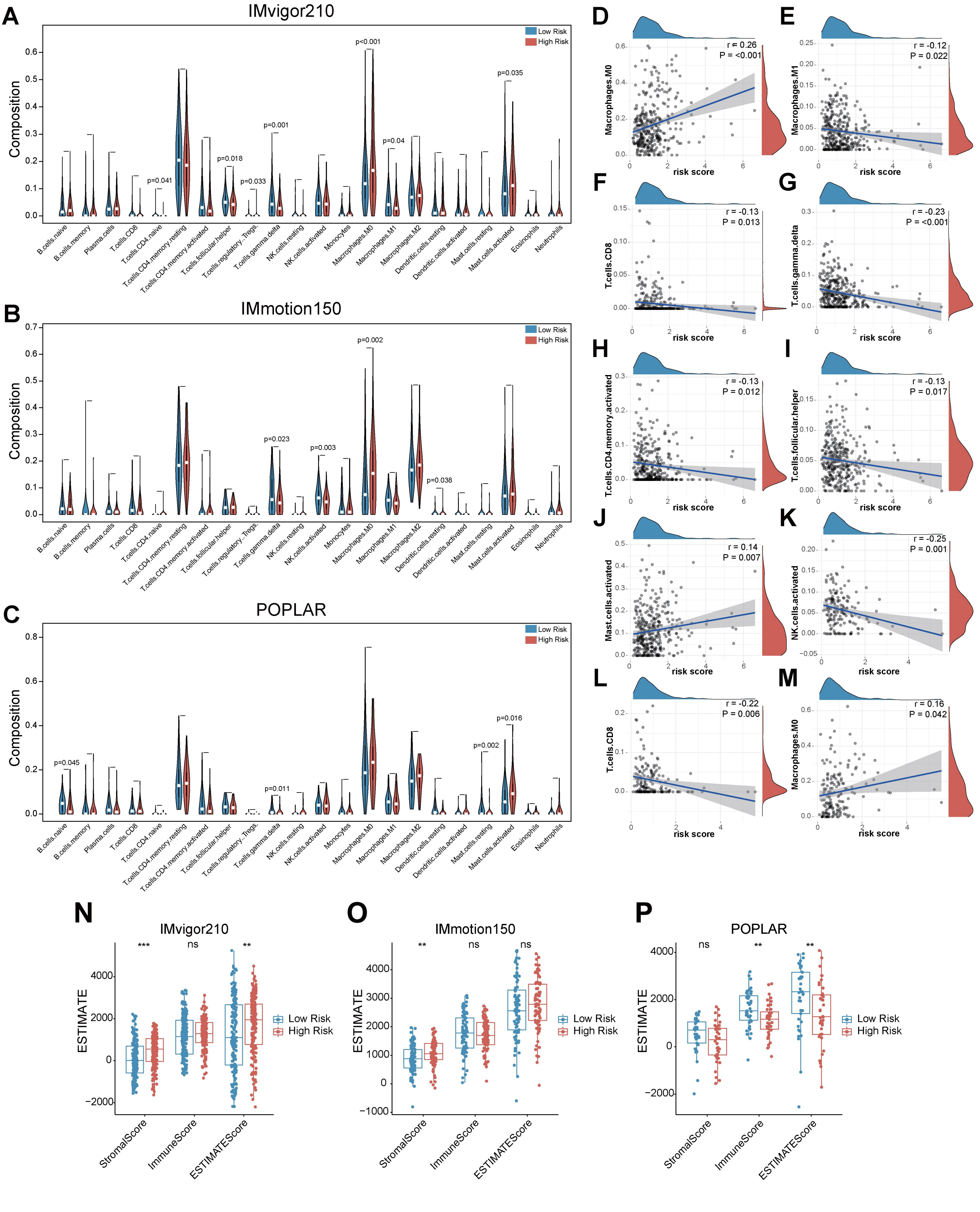

Figure S3. Difference of TME components between high- and low-risk groups. **(A-C)** The infiltrating levels of 22 immune cell types between the high- and low-risk groups in IMvigor210 **(A)**, IMmotion150 **(B)** and POPLAR **(C)**. **(D-M)** The correlation between risk score and the level of infiltrating immune cell in IMvigor210 **(D-J)** and IMmotion150 **(K-M)**. **(N-P)** Comparison of immune, stromal, and ESTIMATE score distribution between high- and low-risk groups in IMvigor210 **(N)**, IMmotion150 **(O)** and POPLAR **(P)**. TME, tumor microenvironment. * $P < 0.05$, ** $P < 0.01$, ***$P < 0.001$, ns no significant.

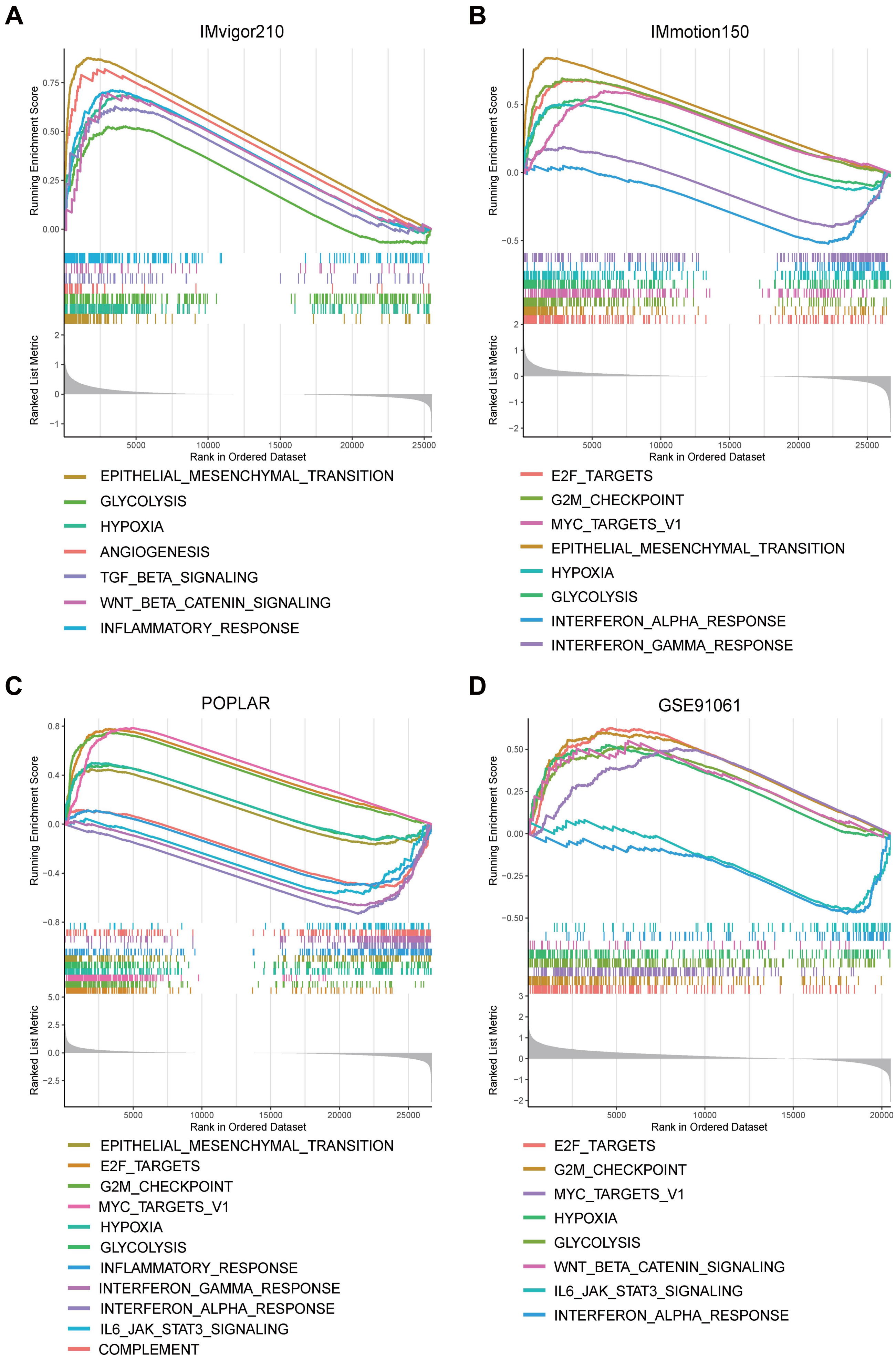

Figure S4. GSEA analysis based the genelist ordered by log2(FoldChange). **(A-D)** GSEA plot for significantly enriched hallmark gene sets associated with the risk score in IMvigor210 **(A)**, IMmotion150 **(B)**, POPLAR **(C)** and GSE91061 **(D)**. GSEA: Gene Set Enrichment Analysis

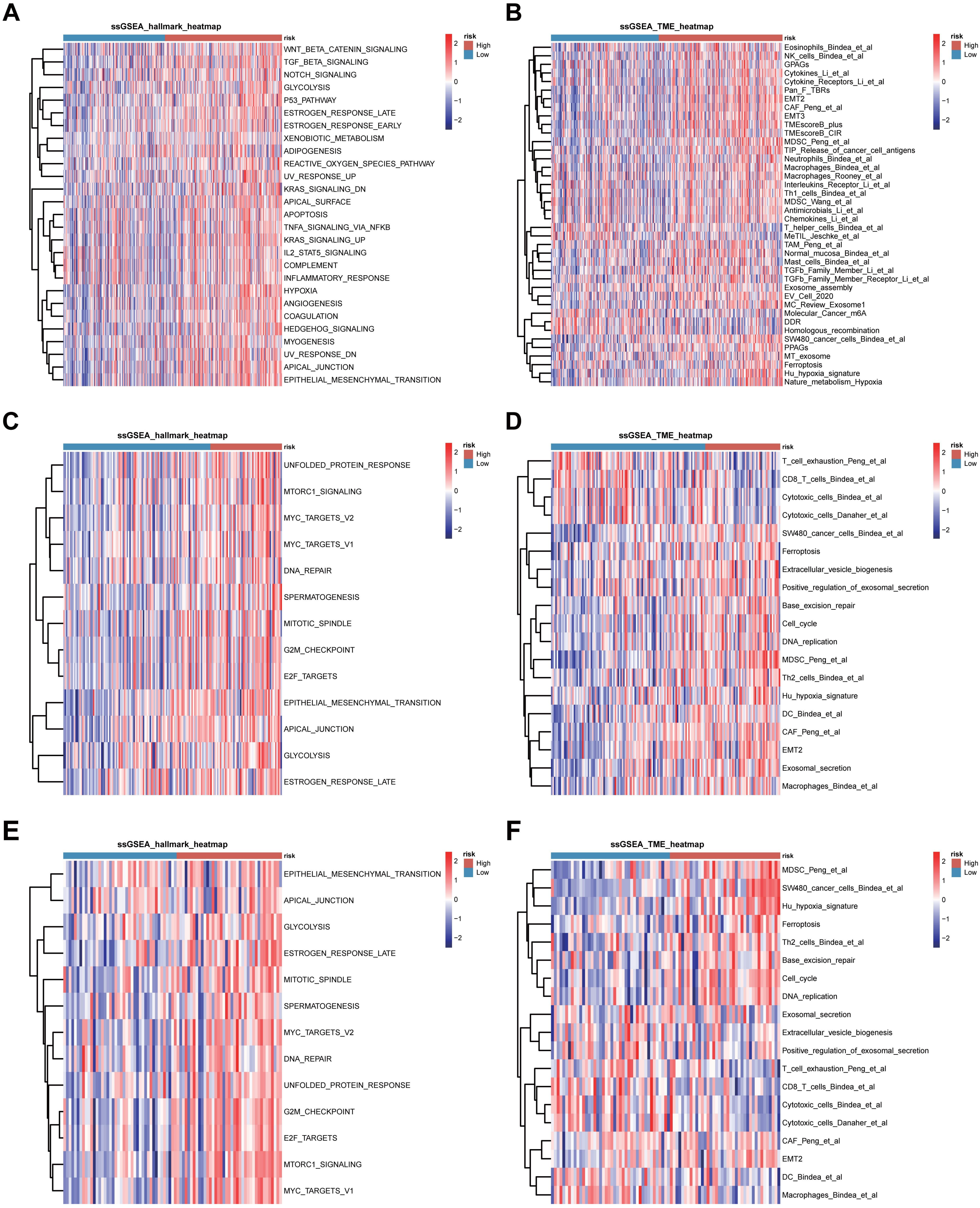

Figure S5. Heatmap of ssGSEA scores of TME and hallmark gene sets. **(A-F)** Heatmap showing the significantly different gene sets of TME (left panel) and hallmark (right panel) gene sets between risk groups in IMvigor210 **(A-B)**, IMmotion150 **(C-D)** and POPLAR **(E-F)**. ssGSEA, single-sample gene set enrichment analysis.

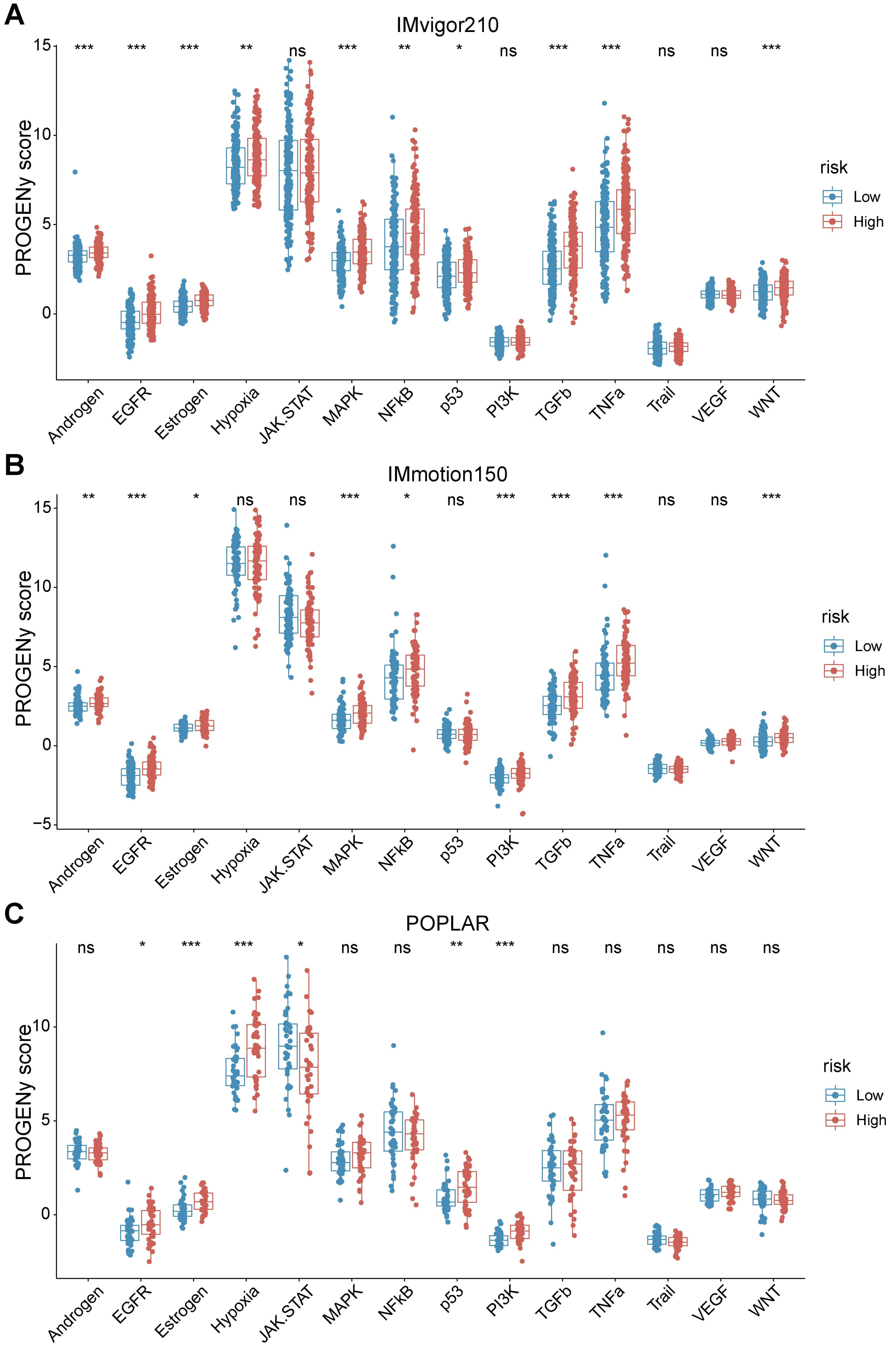

Figure S6. Difference of pathways activity between the high- and low-risk groups. **(A-C)** The pathway activities levels of 14 common malignant signaling pathways between high- and low-risk groups in IMvigor210 **(A)**, IMmotion150 **(B)** and POPLAR **(C)**. * $P < 0.05$, ** $P < 0.01$, ***$P < 0.001$, ns no significant.

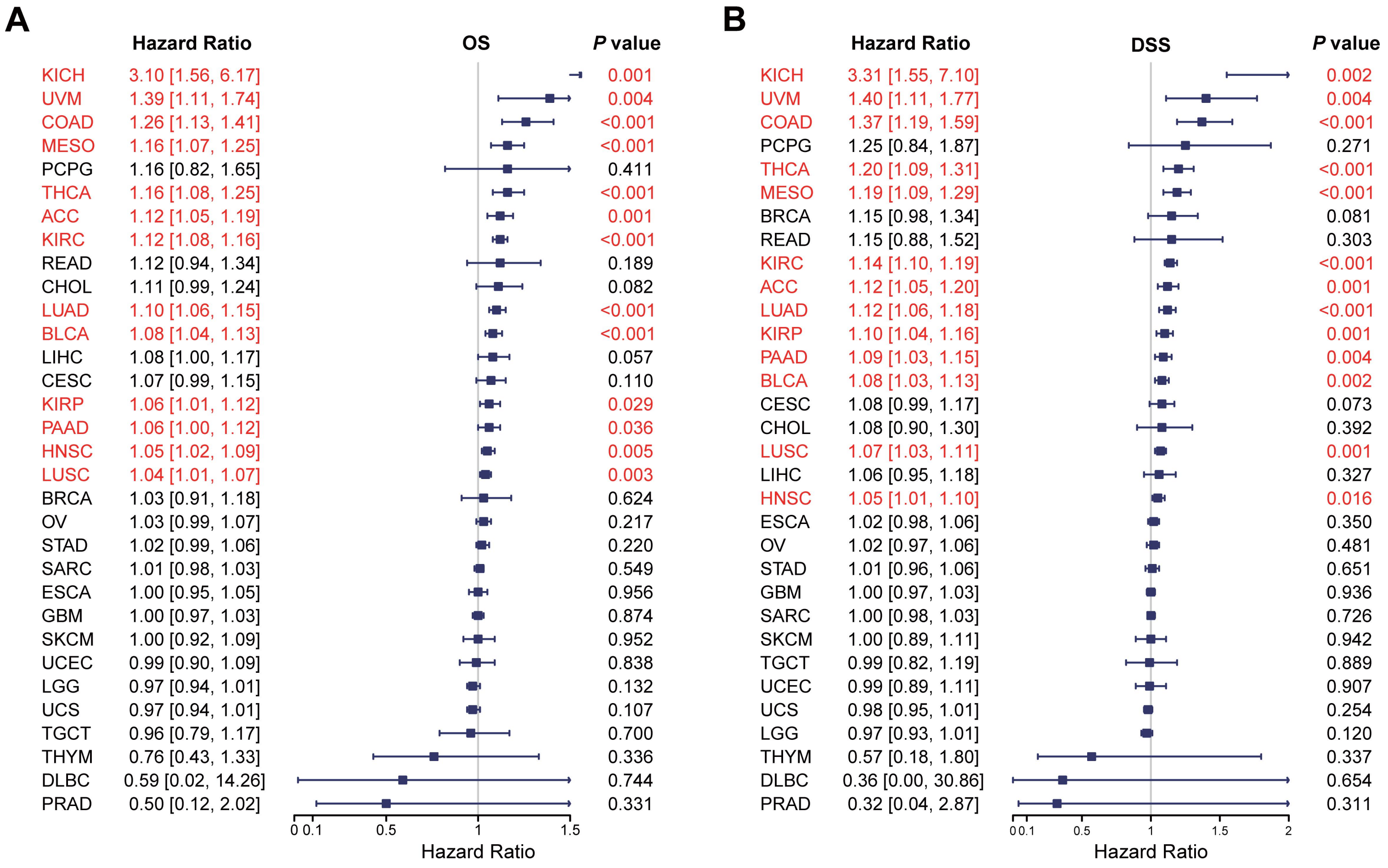

Figure S7. Univariate Cox regression analysis of risk score. **(A)** Forest plots showed the relation between risk score and OS in TCGA pan-cancer. **(B)** Forest plots showed the relation between risk score and DSS in TCGA pan-cancer. Red color represents significant results (P < 0.05). DSS, Disease-specific survival; OS, overall survival; TCGA, The Cancer Genome Atlas. Abbreviation list of tumor cohorts from TCGA is given in **Table S4**.

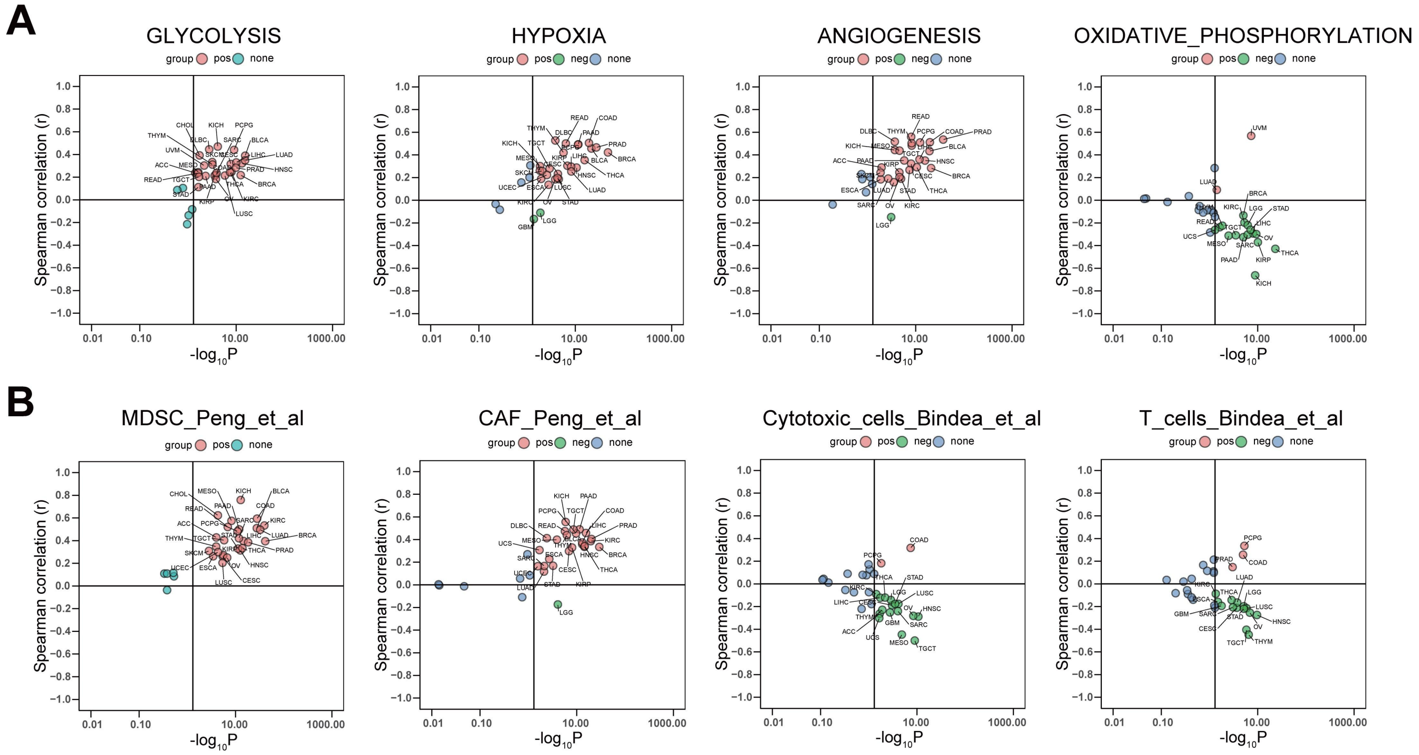

Figure S8. Correlation analysis between risk score and ssGSEA score in in different cancer types from the TCGA dataset. **(A-B)** Correlation analysis between risk score and ssGSEA score of hallmark **(A)** and TME **(B)** in TCGA pan-cancer. ssGSEA, single-sample gene set enrichment analysis; TCGA, The Cancer Genome Atlas. Abbreviation list of tumor cohorts from TCGA is given in **Table S4**.

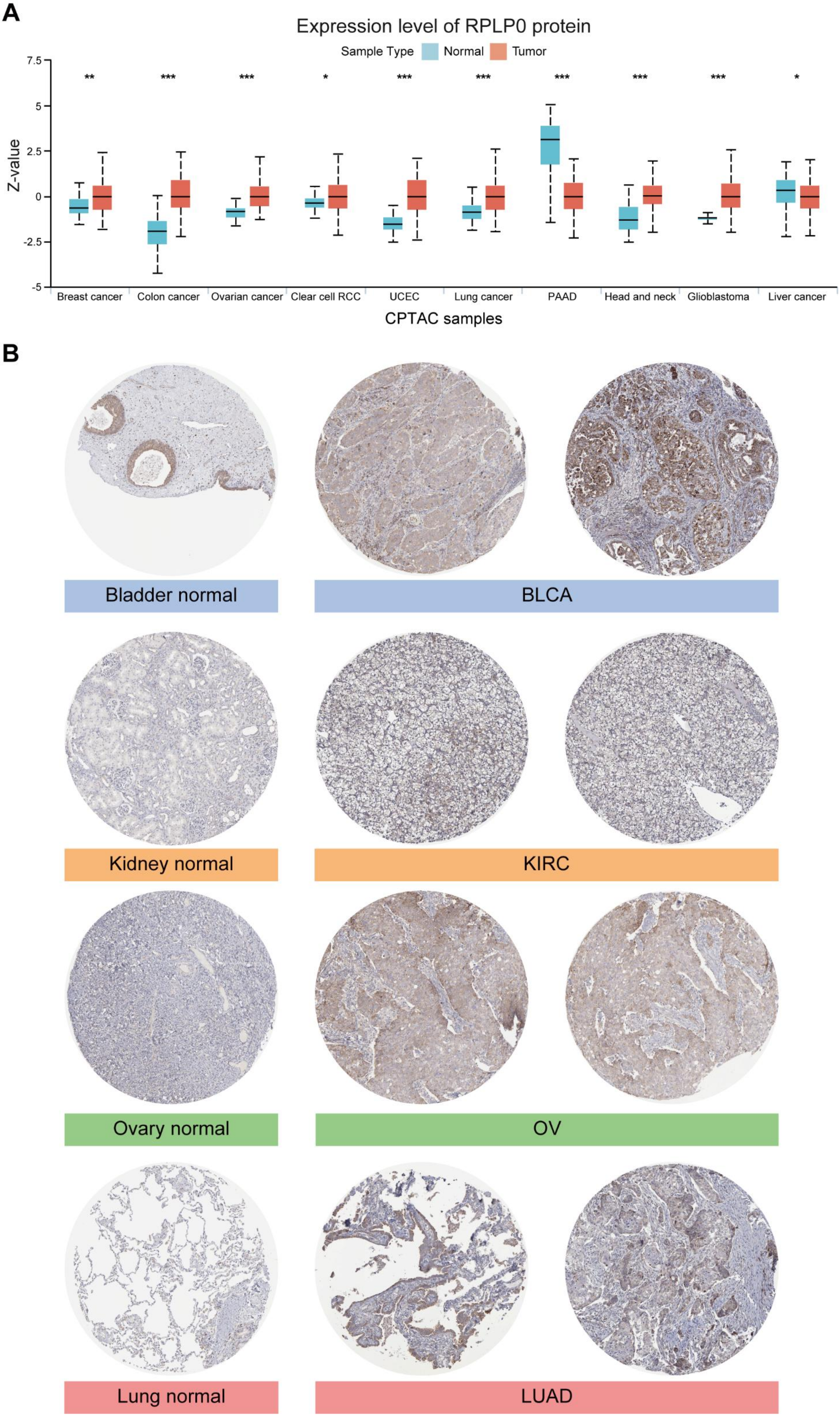
A
Expression level of RPLP0 protein
Sample Type
Normal
Tumor
Z-value
7.5
5
2.5
0
-2.5
-5
**
***
***
*
***
***
***
***
***
*
Breast cancer
Colon cancer
Ovarian cancer
Clear cell RCC
UCEC
Lung cancer
PAAD
Head and neck
Glioblastoma
Liver cancer
CPTAC samples
B
Bladder normal
BLCA
Kidney normal
KIRC
Ovary normal
OV
Lung normal
LUAD

Figure S9.

RPLP0 protein expression profile in normal tissues and tumors.(A) Expression level of RPLP0 protein in TCGA cohorts based on the data from CPTAC database. (B) Images of immunohistochemistry staining of RPLP0 in indicated tumors and normal specimens from the HPA database. CPTAC, Clinical Proteomic Tumor Analysis Consortium ;HPA, the Human Protein Atlas; TCGA, The Cancer Genome Atlas. Abbreviation list of tumor cohorts from TCGA is given in **Table S4**. * P < 0.05, ** P < 0.01, ***P < 0.001.

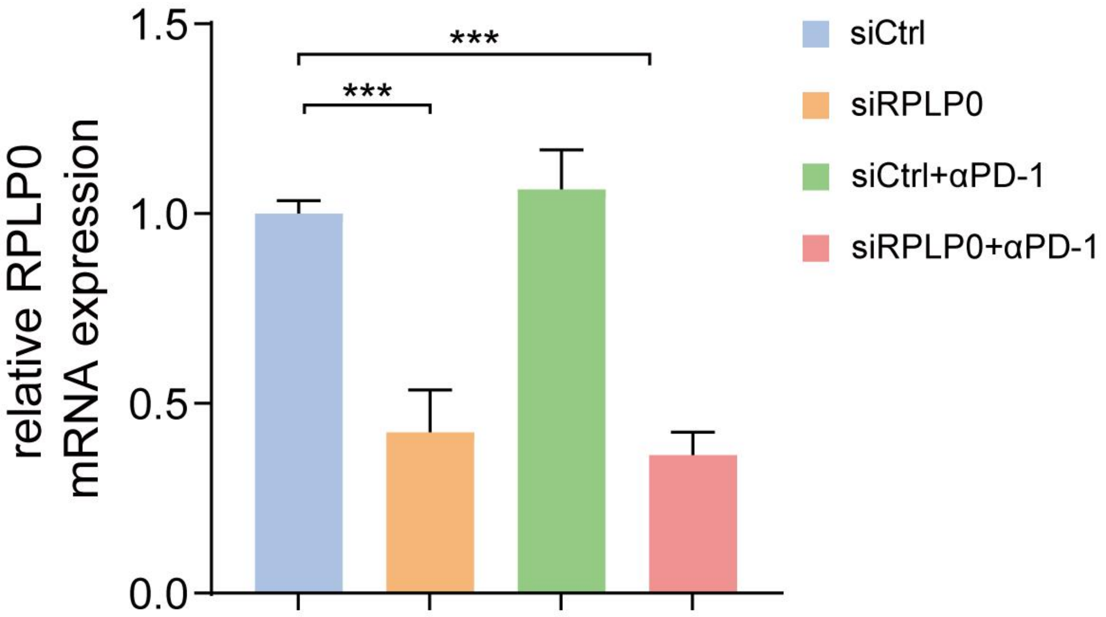

Figure S10.

Bar charts representing the mRNA expression level of RPLP0 in tumor tissues from mice in indicated groups (n = 5 per group). One-way ANOVA followed by Dunnett's multiple comparisons tests were used to compare data in four groups. * P < 0.05, ** P < 0.01, ***P < 0.001.

Table S1: Patients clinical characteristics

Indication	**IMvigor210**	**POPLAR**	**IMmotion150**	**IMmotion151**	**CheckMate**	**GSE91061**
Sample count	348	81	162	407	170	51
Arm	Atezolizumab	Atezolizumab	Atezolizumab (n=77)	Atezolizumab+ Bevacizumab	Nivolumab	Nivolumab
			Atezolizumab+ Bevacizumab (n=85)			
Gender Male (%)	272 (78.2)	56 (69.1)	118 (72.8)	281 (69.0)	128 (75.3)	NA
Age >=60 years (%)	NA	47 (58.0)	92 (56.8)	243 (59.7)	101 (59.8)	NA
Samples with WES	234	50	107	341	112	46
TMB (muts/Mb), median (IQR)	8.0 (9.0)	15.5 (13.2)	9.0 (6.4)	3.5 (2.6)	58 (33.5)	182.5 (361.5)
ORR, CR/PR, n(%)	68 (19.5)	47 (58.0)	102 (63.0)	150 (39.5)	37 (23.0)	10 (20.4)
OS (months), median (IQR)	8.0 (14.9)	12.6 (13.1)	NA	NA	22.8 (40.5)	16.1 (20.5)
PFS (months), median (IQR)	NA	2.8 (7.0)	8.1 (15.9)	9.7 (11.3)	3.8 (7.6)	NA

Table S2: Oligo sequences used in quantitative real-time PCR

Taget	Primer(5' - 3')	
ACTB (human)	Forward	CATGTACGTTGCTATCCAGGC
	Reverse	CTCCTTAATGTCACGCACGAT
RPLP0 (human)	Forward	TGGCAGCATCTACAACCCTG
	Reverse	GACAAGGCCAGGACTCGTTT
Actb (mouse)	Forward	GGCTGTATTCCCCTCCATCG
	Reverse	CCAGTTGGTAACAATGCCATGT
Rplp0 (mouse)	Forward	TGAGATTCGGGATATGCTGTTGG
	Reverse	CGGGTCCTAGACCAGTGTTCT

Table S3: siRNA target sequence used for gene knockdown

Name	Sequence (5' - 3')
Negative control siRNA	UUCUCCGAACGUGUCACGUTT
si-*Rplp0*	CGGAGGAAUCAGAUGAGGAUA

Table S4: Abbreviation of 32 TCGA cancer types

Abbreviation	Detail
ACC	Adrenocortical carcinoma
BLCA	Bladder Urothelial Carcinoma
BRCA	Breast invasive carcinoma
CESC	Cervical squamous cell carcinoma and endocervical adenocarcinoma
CHOL	Cholangio carcinoma
COAD	Colon adenocarcinoma
DLBC	Lymphoid Neoplasm Diffuse Large B-cell Lymphoma
ESCA	Esophageal carcinoma
GBM	Glioblastoma multiforme
HNSC	Head and Neck squamous cell carcinoma
KICH	Kidney Chromophobe
KIRC	Kidney renal clear cell carcinoma
KIRP	Kidney renal papillary cell carcinoma
LGG	Brain Lower Grade Glioma
LIHC	Liver hepatocellular carcinoma
LUAD	Lung adenocarcinoma
LUSC	Lung squamous cell carcinoma
MESO	Mesothelioma
OV	Ovarian serous cystadenocarcinoma
PAAD	Pancreatic adenocarcinoma
PCPG	Pheochromocytoma and Paraganglioma
PRAD	Prostate adenocarcinoma
READ	Rectum adenocarcinoma
SARC	Sarcoma
SKCM	Skin Cutaneous Melanoma
STAD	Stomach adenocarcinoma
TGCT	Testicular Germ Cell Tumors
THCA	Thyroid carcinoma
THYM	Thymoma
UCEC	Uterine Corpus Endometrial Carcinoma
UCS	Uterine Carcinosarcoma
UVM	Uveal Melanoma

Table S5: Details of 304 immunosuppression-related genes (ISRGs)

Symbol	Ensemble ID	Entrez ID	SeqNames	Length	N_Exons
ABCC1	ENSG00000103222.17	4363	chr16	8677	34
ABLIM2	ENSG00000163995.17	84448	chr4	6303	33
ADAM7	ENSG00000069206.14	8756	chr8	5226	27
ADAT2	ENSG00000189007.14	134637	chr6	7493	7
ADCY7	ENSG00000121281.11	113	chr16	9749	36
ADH1B	ENSG00000196616.11	125	chr4	6534	14
ADORA1	ENSG00000163485.14	134	chr1	6625	11
AHCY	ENSG00000101444.11	191	chr20	4065	14
AHCYL2	ENSG00000158467.15	23382	chr7	5887	20
ANKRD2	ENSG00000165887.10	26287	chr10	4370	26
ANKRD7	ENSG00000106013.11	56311	chr7	1358	7
ANXA13	ENSG00000104537.15	312	chr8	1593	12
APLP2	ENSG00000084234.15	334	chr11	7415	35
ARAP1	ENSG00000186635.13	116985	chr11	5646	36
ARHGEF4	ENSG00000136002.15	50649	chr2	18645	34
ARL10	ENSG00000175414.6	285598	chr5	6194	8
ARL4D	ENSG00000175906.4	379	chr17	1585	2
ASGR2	ENSG00000161944.15	433	chr17	3292	19
ATP1B3	ENSG00000069849.9	483	chr3	1853	7
ATP6V0D2	ENSG00000147614.3	245972	chr8	2370	8
AVEN	ENSG00000169857.6	57099	chr15	1555	7
B4GALNT3	ENSG00000139044.9	283358	chr12	3438	20
BAAT	ENSG00000136881.10	570	chr9	3528	5
BLOC1S4	ENSG00000186222.4	55330	chr4	1546	1
BNC1	ENSG00000169594.11	646	chr15	9875	12
BTBD19	ENSG00000222009.7	149478	chr1	7077	22
C10orf62	ENSG00000203942.4	414157	chr10	1242	1
C10orf90	ENSG00000154493.16	118611	chr10	6222	17
C12orf54	ENSG00000177627.8	121273	chr12	4543	53
C1S	ENSG00000182326.13	716	chr12	2984	14
C3orf18	ENSG00000088543.13	51161	chr3	5047	11
C8B	ENSG00000021852.11	732	chr1	3025	18
CACNA2D4	ENSG00000151062.13	93589	chr12	10609	75
CAPN2	ENSG00000162909.16	824	chr1	3727	22
CARM1	ENSG00000142453.10	10498	chr19	4677	18
CASP8	ENSG00000064012.20	841	chr2	16094	78
CBR1	ENSG00000159228.11	873	chr21	2684	4
CCBE1	ENSG00000183287.12	147372	chr18	9056	17
CCDC102A	ENSG00000135736.5	92922	chr16	2429	9
CCDC80	ENSG00000091986.14	151887	chr3	7342	11
CCL24	ENSG00000106178.5	6369	chr7	360	3
CD1A	ENSG00000158477.6	909	chr1	2096	6
CD276	ENSG00000103855.16	80381	chr15	5266	13
CD320	ENSG00000167775.9	51293	chr19	1251	5
CDA	ENSG00000158825.5	978	chr1	960	4
CDCP1	ENSG00000163814.6	64866	chr3	10414	13
CELA1	ENSG00000139610.1	1990	chr12	952	8
CEP41	ENSG00000106477.17	95681	chr7	15870	23
CFH	ENSG00000000971.14	3075	chr1	4627	26
CFHR1	ENSG00000244414.5	3078	chr1	1492	8
CFHR3	ENSG00000116785.12	10878	chr1	4603	17
CFHR4	ENSG00000134365.11	10877	chr1	3770	19
CFI	ENSG00000205403.11	3426	chr4	2705	17
CHRNB4	ENSG00000117971.10	1143	chr15	3446	7
CHST3	ENSG00000122863.5	9469	chr10	13666	5

Symbol	Ensemble ID	Entrez ID	SeqNames	Length	N_Exons
CHST6	ENSG00000183196.7	4166	chr16	9422	5
CLEC11A	ENSG00000105472.11	6320	chr19	1407	4
CLEC5A	ENSG00000258227.5	23601	chr7	8514	14
CLIP4	ENSG00000115295.18	79745	chr2	10850	39
CNN2	ENSG00000064666.13	1265	chr19	2458	7
COL16A1	ENSG00000084636.16	1307	chr1	6264	73
COL18A1	ENSG00000182871.13	80781	chr21	6058	43
COMMD10	ENSG00000145781.7	51397	chr5	1424	7
CORO2B	ENSG00000103647.11	10391	chr15	4410	16
CPEB1	ENSG00000214575.8	64506	chr15	6874	22
CRACR2A	ENSG00000130038.8	84766	chr12	7005	25
CRB2	ENSG00000148204.10	286204	chr9	9771	23
CRTAC1	ENSG00000095713.12	55118	chr10	3532	18
CRYAB	ENSG00000109846.6	1410	chr11	2610	10
CSTA	ENSG00000121552.3	1475	chr3	828	3
CYP19A1	ENSG00000137869.12	1588	chr15	6618	16
CYP2C18	ENSG00000108242.11	1562	chr10	2545	9
CYP2C19	ENSG00000165841.8	1557	chr10	1473	9
CYP2C9	ENSG00000138109.9	1559	chr10	2153	11
CYP8B1	ENSG00000180432.5	1582	chr3	3950	1
CYS1	ENSG00000205795.4	192668	chr2	2719	3
DCAF4L1	ENSG00000182308.6	285429	chr4	4772	1
DCLK1	ENSG00000133083.13	9201	chr13	25919	24
DGAT1	ENSG00000185000.8	8694	chr8	3721	17
DHRS9	ENSG00000073737.15	10170	chr2	5822	19
ECI2	ENSG00000198721.11	10455	chr6	3286	25
EEF1D	ENSG00000104529.16	1936	chr8	29196	136
EIF3B	ENSG00000106263.16	8662	chr7	6093	38
EIF3D	ENSG00000100353.16	8664	chr22	1949	15
EIF3H	ENSG00000147677.9	8667	chr8	1275	8
EPHX3	ENSG00000105131.6	79852	chr19	2989	11
ESPNL	ENSG00000144488.13	339768	chr2	4967	12
EVA1A	ENSG00000115363.12	84141	chr2	2102	5
EYA4	ENSG00000112319.16	2070	chr6	20874	65
FAM78B	ENSG00000188859.6	149297	chr1	1481	2
FBXO2	ENSG00000116661.9	26232	chr1	3153	13
FCER1A	ENSG00000179639.9	2205	chr1	1172	7
FCGR3B	ENSG00000162747.8	2215	chr1	3101	10
FCGRT	ENSG00000104870.11	2217	chr19	3556	13
FCN2	ENSG00000160339.14	2220	chr9	1663	10
FGA	ENSG00000171560.13	2243	chr4	5297	7
FGF11	ENSG00000161958.9	2256	chr17	2715	5
FGL1	ENSG00000104760.15	2267	chr8	2722	14
FLNC	ENSG00000128591.14	2318	chr7	10065	50
FMOD	ENSG00000122176.10	2331	chr1	5528	5
FOLR2	ENSG00000165457.12	2350	chr11	1519	8
FOXA2	ENSG00000125798.13	3170	chr20	4689	6
GBGT1	ENSG00000148288.10	26301	chr9	5045	12
GEMIN5	ENSG00000082516.8	25929	chr5	5521	29
GFPT2	ENSG00000131459.11	9945	chr5	4048	21
GLI1	ENSG00000111087.8	2735	chr12	4075	16
GLI2	ENSG00000074047.19	2736	chr2	11341	17
GLRX3	ENSG00000108010.10	10539	chr10	3254	27
GNAL	ENSG00000141404.14	2774	chr18	18016	36
GOLGA8O	ENSG00000206127.9	728047	chr15	8967	21
GSTP1	ENSG00000084207.14	2950	chr11	958	7
HDAC10	ENSG00000100429.16	83933	chr22	2707	20

Symbol	Ensemble ID	Entrez ID	SeqNames	Length	N_Exons
HHIPL2	ENSG00000143512.11	79802	chr1	2574	9
HOXA11	ENSG00000005073.5	3207	chr7	2653	2
HOXA3	ENSG00000105997.21	3200	chr7	6645	11
HR	ENSG00000168453.13	55806	chr8	6310	21
HSPA1A	ENSG00000204389.9	3303	chr6	2429	1
HSPA1B	ENSG00000204388.6	3304	chr6	2520	1
HSPD1	ENSG00000144381.15	3329	chr2	2659	14
HTR7	ENSG00000148680.14	3363	chr10	7530	7
IFI27L2	ENSG00000119632.3	83982	chr14	467	4
IFNE	ENSG00000184995.7	338376	chr9	1475	1
IGFBP1	ENSG00000146678.8	3484	chr7	1650	4
IL18	ENSG00000150782.10	3606	chr11	1147	6
IL6R	ENSG00000160712.11	3570	chr1	16064	19
IMPDH1	ENSG00000106348.15	3614	chr7	5523	29
IRS2	ENSG00000185950.8	8660	chr13	6998	2
IRX6	ENSG00000159387.7	79190	chr16	3432	8
ISLR	ENSG00000129009.11	3671	chr15	2385	3
IYD	ENSG00000009765.13	389434	chr6	23478	23
KATNB1	ENSG00000140854.11	10300	chr16	4249	27
KIF6	ENSG00000164627.16	221458	chr6	4603	26
KLHL17	ENSG00000187961.12	339451	chr1	6427	30
KLHL21	ENSG00000162413.15	9903	chr1	6034	6
L3MBTL3	ENSG00000198945.6	84456	chr6	6498	29
LAMB1	ENSG00000091136.12	3912	chr7	5846	34
LRRN4	ENSG00000125872.7	164312	chr20	2955	5
LY6E	ENSG00000160932.9	4061	chr8	1280	5
LY6K	ENSG00000160886.12	54742	chr8	5289	8
LZTS2	ENSG00000107816.16	84445	chr10	3454	8
MAP4K4	ENSG00000071054.14	9448	chr2	9837	41
MARCO	ENSG00000019169.10	8685	chr2	1835	17
MARS2	ENSG00000247626.4	92935	chr2	3087	1
MATN3	ENSG00000132031.11	4148	chr2	2583	8
MCTP1	ENSG00000175471.18	79772	chr5	22366	81
MEIOB	ENSG00000162039.13	254528	chr16	2372	16
METTL8	ENSG00000123600.17	79828	chr2	9622	18
MGMT	ENSG00000170430.9	4255	chr10	1900	9
MLLT11	ENSG00000213190.3	10962	chr1	2158	2
MMADHC	ENSG00000168288.11	27249	chr2	1437	8
MMP7	ENSG00000137673.7	4316	chr11	1119	6
MRPS27	ENSG00000113048.15	23107	chr5	2900	13
MYBPC2	ENSG00000086967.9	4606	chr19	3598	28
NBL1	ENSG00000158747.12	4681	chr1	3620	12
NCKAP5L	ENSG00000167566.15	57701	chr12	5544	19
NEFM	ENSG00000104722.12	4741	chr8	3627	4
NKAIN4	ENSG00000101198.13	128414	chr20	2517	10
NLRP1	ENSG00000091592.14	22861	chr17	6180	19
NOP56	ENSG00000101361.13	10528	chr20	4206	24
NPAS2	ENSG00000170485.15	4862	chr2	8660	31
NRBP2	ENSG00000185189.14	340371	chr8	8574	29
OBSL1	ENSG00000124006.13	23363	chr2	15986	32
OSM	ENSG00000099985.3	5008	chr22	3855	5
OXT	ENSG00000101405.3	5020	chr20	513	3
PARVB	ENSG00000188677.13	29780	chr22	2964	23
PAX8	ENSG00000125618.15	7849	chr2	6778	14
PCDHA10	ENSG00000250120.5	56139	chr5	12752	9
PDCD4	ENSG00000150593.14	27250	chr10	5737	22
PDE11A	ENSG00000128655.15	50940	chr2	10019	26

Symbol	Ensemble ID	Entrez ID	SeqNames	Length	N_Exons
PHACTR3	ENSG00000087495.15	116154	chr20	3541	18
PI15	ENSG00000137558.6	51050	chr8	8091	10
PID1	ENSG00000153823.17	55022	chr2	2822	4
PILRB	ENSG00000121716.17	29990	chr7	1438	4
PIP5KL1	ENSG00000167103.10	138429	chr9	3532	15
PKNOX2	ENSG00000165495.14	63876	chr11	4140	18
PLA2G12B	ENSG00000138308.5	84647	chr10	3942	9
PLA2G2A	ENSG00000188257.9	5320	chr1	1258	8
PLA2G4E	ENSG00000188089.12	123745	chr15	4779	20
PLP2	ENSG00000102007.9	5355	chrX	1049	5
PLTP	ENSG00000100979.13	5360	chr20	3352	20
POU5F1B	ENSG00000212993.4	5462	chr8	1599	1
POU5F2	ENSG00000248483.5	134187	chr5	1295	1
POU6F2	ENSG00000106536.18	11281	chr7	2826	12
PPP1R14B	ENSG00000173457.9	26472	chr11	987	4
PPP4R4	ENSG00000119698.10	57718	chr14	17373	107
PRAM1	ENSG00000133246.10	84106	chr19	2602	12
PRSS3	ENSG00000010438.15	5646	chr9	1558	10
PSMD14	ENSG00000115233.10	10213	chr2	1717	12
PTCD1	ENSG00000106246.16	26024	chr7	5505	8
PTGIR	ENSG00000160013.7	5739	chr19	5583	11
PYGB	ENSG00000100994.10	5834	chr20	4731	23
RAC2	ENSG00000128340.13	5880	chr22	2172	13
RAD51AP2	ENSG00000214842.5	729475	chr2	7450	8
RASSF9	ENSG00000198774.4	9182	chr12	1796	2
RHBDF1	ENSG00000007384.14	64285	chr16	6536	43
RNF169	ENSG00000166439.5	254225	chr11	7823	6
RNF207	ENSG00000158286.11	388591	chr1	7083	31
RPL13A	ENSG00000142541.15	23521	chr19	2517	18
RPL18	ENSG00000063177.11	6141	chr19	1766	14
RPL18A	ENSG00000105640.11	6142	chr19	671	5
RPL19	ENSG00000108298.8	6143	chr17	1260	8
RPL27	ENSG00000131469.11	6155	chr17	495	5
RPL28	ENSG00000108107.11	6158	chr19	10106	10
RPL4	ENSG00000174444.13	6124	chr15	1441	10
RPLP0	ENSG00000089157.14	6175	chr12	1366	9
RPLP1	ENSG00000137818.10	6176	chr15	512	4
RPS18	ENSG00000231500.5	6222	chr6	542	6
RPS19	ENSG00000105372.5	6223	chr19	849	6
RPS2	ENSG00000140988.14	6187	chr16	942	7
RPS23	ENSG00000186468.11	6228	chr5	3314	4
RPS24	ENSG00000138326.17	6229	chr10	9349	45
RPS27A	ENSG00000143947.11	6233	chr2	1469	8
RPS28	ENSG00000233927.4	6234	chr19	382	4
RPS3	ENSG00000149273.13	6188	chr11	7592	27
RPS5	ENSG00000083845.7	6193	chr19	741	6
RPS8	ENSG00000142937.10	6202	chr1	705	6
RPS9	ENSG00000170889.12	6203	chr19	35697	220
RRP12	ENSG00000052749.12	23223	chr10	4426	34
RRP8	ENSG00000132275.9	23378	chr11	1751	7
S100A1	ENSG00000160678.10	6271	chr1	594	3
S100A9	ENSG00000163220.10	6280	chr1	577	3
SAA1	ENSG00000173432.9	6288	chr11	775	6
SBSN	ENSG00000189001.9	374897	chr19	2665	6
SDCBP2	ENSG00000125775.13	27111	chr20	1955	11
SEMA7A	ENSG00000138623.8	8482	chr15	3780	17
SERPINA3	ENSG00000273259.2	12	chr14	1590	5

Symbol	Ensemble ID	Entrez ID	SeqNames	Length	N_Exons
SERPINA5	ENSG00000188488.12	5104	chr14	2337	6
SERPINB4	ENSG00000206073.9	6318	chr18	3426	16
SERPINE2	ENSG00000135919.11	5270	chr2	6558	25
SERPINF2	ENSG00000167711.12	5345	chr17	5573	29
SERPINH1	ENSG00000149257.12	871	chr11	5908	10
SHANK1	ENSG00000161681.14	50944	chr19	10491	28
SHH	ENSG00000164690.6	6469	chr7	1576	3
SIGLEC12	ENSG00000254521.5	89858	chr19	2732	9
SIX1	ENSG00000126778.8	6495	chr14	2687	2
SIX5	ENSG00000177045.7	147912	chr19	3331	3
SLAMF9	ENSG00000162723.8	89886	chr1	1740	6
SLC11A1	ENSG00000018280.15	6556	chr2	11886	38
SLC18A2	ENSG00000165646.10	6571	chr10	3872	16
SLC25A3	ENSG00000075415.11	5250	chr12	2314	10
SLC38A5	ENSG00000017483.13	92745	chrX	5360	42
SLC39A13	ENSG00000165915.12	91252	chr11	13614	58
SLC39A4	ENSG00000147804.8	55630	chr8	3407	16
SLC3A2	ENSG00000168003.15	6520	chr11	4687	23
SLC6A20	ENSG00000163817.14	54716	chr3	5952	13
SLC7A5	ENSG00000103257.7	8140	chr16	4679	13
SLPI	ENSG00000124107.5	6590	chr20	596	4
SNAI1	ENSG00000124216.3	6615	chr20	1709	3
SNRPE	ENSG00000182004.11	6635	chr1	1547	5
SPDYE4	ENSG00000183318.10	388333	chr17	3353	20
SPIN2A	ENSG00000147059.8	54466	chrX	5045	10
SQLE	ENSG00000104549.10	6713	chr8	2989	11
SRPX	ENSG00000101955.13	8406	chrX	2463	11
SRXN1	ENSG00000271303.1	140809	chr20	2698	2
SSTR5	ENSG00000162009.8	6755	chr16	7991	5
SULT1A1	ENSG00000196502.10	6817	chr16	4065	19
SYT12	ENSG00000173227.12	91683	chr11	3820	11
SYT13	ENSG00000019505.6	57586	chr11	9630	11
TAF1D	ENSG00000166012.13	79101	chr11	1312	6
TAS1R2	ENSG00000179002.5	80834	chr1	2521	6
TBC1D2	ENSG00000095383.18	55357	chr9	7091	27
TBX15	ENSG00000092607.12	6913	chr1	6938	12
TBX18	ENSG00000112837.15	9096	chr6	7046	10
TCIRG1	ENSG00000110719.8	10312	chr11	9804	51
TCN1	ENSG00000134827.6	6947	chr11	1567	9
TENM2	ENSG00000145934.14	57451	chr5	11410	38
TFB2M	ENSG00000162851.7	64216	chr1	1786	8
TFE3	ENSG00000068323.15	7030	chrX	4462	14
TGFB1I1	ENSG00000140682.17	7041	chr16	1982	14
THBS2	ENSG00000186340.13	7058	chr6	5881	23
TICAM1	ENSG00000127666.9	148022	chr19	2722	2
TIGD6	ENSG00000164296.6	81789	chr5	4682	3
TLR2	ENSG00000137462.6	7097	chr4	7460	12
TM4SF19	ENSG00000145107.14	116211	chr3	1677	7
TM4SF4	ENSG00000169903.6	7104	chr3	1607	5
TMEM179	ENSG00000258986.5	388021	chr14	3652	5
TMEM59L	ENSG00000105696.7	25789	chr19	1970	10
TNFSF14	ENSG00000125735.9	8740	chr19	7659	8
TNNI1	ENSG00000159173.17	7135	chr1	6143	9
TNNT2	ENSG00000118194.17	7139	chr1	2035	25
TP63	ENSG00000073282.11	8626	chr3	14539	22
TRIM29	ENSG00000137699.15	23650	chr11	4183	12
TSHZ3	ENSG00000121297.6	57616	chr19	4913	2

Symbol	Ensemble ID	Entrez ID	SeqNames	Length	N_Exons
TUBA1C	ENSG00000167553.13	84790	chr12	1553	4
TUBA3E	ENSG00000152086.8	112714	chr2	1540	5
UBTD1	ENSG00000165886.4	80019	chr10	1557	3
UCHL3	ENSG00000118939.16	7347	chr13	1850	15
UCN2	ENSG00000145040.3	90226	chr3	1485	2
UGT3A1	ENSG00000145626.10	133688	chr5	7667	17
UNC45A	ENSG00000140553.15	55898	chr15	4800	27
UST	ENSG00000111962.7	10090	chr6	5731	15
VCX3B	ENSG00000205642.8	425054	chrX	1175	3
VENTX	ENSG00000151650.7	27287	chr10	2412	3
VSTM1	ENSG00000189068.8	284415	chr19	9457	91
WDFY2	ENSG00000139668.8	115825	chr13	6371	24
WNT3	ENSG00000108379.8	7473	chr17	3355	5
WT1	ENSG00000184937.11	7490	chr11	3399	12
ZDHHC12	ENSG00000160446.17	84885	chr9	1891	7
ZFP92	ENSG00000189420.8	139735	chrX	7395	8
ZFYVE27	ENSG00000155256.16	118813	chr10	7821	23
ZNF20	ENSG00000132010.14	7568	chr19	3188	5
ZNF692	ENSG00000171163.14	55657	chr1	3215	18
ZSCAN12	ENSG00000158691.13	9753	chr6	18297	26
ZYX	ENSG00000159840.14	7791	chr7	2691	12

Table S6: Univariate Cox regression of commonly used characteristics and risk score in immune checkpoint inhibitor (ICI) treatment sets

IMvigor210 (OS)			
**variable**	**HR**	**95% CI**	***P* value**
**Sex**			
Female	1		
Male	0.785	0.583-1.059	0.113
**IC level[1]**			
IC0	1		
IC1	0.872	0.642-1.185	0.383
IC2	0.566	0.405-0.780	<0.001
**TC level[2]**			
IC0	1		
TC1	1.111	0.656-1.882	0.694
TC2	0.992	0.691- 1.425	0.967
**PDCD1**	0.789	0.645-0.966	0.021
**CD274**	0.869	0.752-1.003	0.055
**TMB**	0.956	0.583-0.980	<0.001
**risk score**	1.331	1.194-1.484	<0.001

POPLAR (OS)			
**variable**	**HR**	**95% CI**	***P* value**
**Age**			
<=60	1		
>60	0.875	0.504-1.518	0.635
**Sex**			
Female	1		
Male	1.063	0.587-1.925	0.84
**IC level**			
IC0	1		
IC1	0.606	0.332-1.108	0.103
IC2	0.571	0.232-1.406	0.223
IC3	0.431	0.101-1.833	0.254
**TC level**			
TC0	1		
TC1	0.847	0.329-2.182	0.731
TC2	1.061	0.417-2.7	0.901
TC3	0.343	0.106-1.114	0.075
**PDCD1**	0.611	0.409-0.911	0.016
**CD274**	0.803	0.618-1.042	0.099
**TMB**	1.005	1.001-1.008	0.006
**risk score**	1.239	1.114-1.379	<0.001

IMmotion150 (PFS)			
**variable**	**HR**	**95% CI**	***P* value**
**Age**			
<=60	1		
>60	0.777	0.54- 1.118	0.174
**Sex**			
Female	1		
Male	1.029	0.685- 1.546	0.891
**IC level**			
IC0	1		
IC1	0.778	0.517-1.171	0.229
IC2	1.032	0.588-1.811	0.913
IC3	1.013	0.480-2.137	0.973
**TC level**			
TC0	1		
TC1	1.093	0.552-2.166	0.798
TC2	2.108	0.917-4.845	0.079
TC3	0.952	0.387-2.340	0.915
**PDCD1**	0.777	0.629-0.960	0.02
**CD274**	0.871	0.708-1.071	0.19
**TMB**	1.005	0.999-1.012	0.113
**risk score**	1.631	1.340-1.985	<0.001

[1] PD-L1 expression on immune cells measure by immunohistochemistry
[2] PD-L1 expression on tumor cells measured by immunohistochemistry

Supplementary Method

Genomic and clinical data sources and preprocessing

RNA-seq data obtained before immunotherapy and matched clinical data of patients who received anti-PD1/PDL1 immunotherapy in several cohorts across multiple cancer types and other public data analyzed in this work were collected.

① RNA-seq data in Transcripts Per Million (TPM) and matched clinical data of patients with metastatic urothelial cancer (mUC) treated with atezolizumab (anti-PD-L1 antibody) were retrieved from the R package IMvigor210CoreBiologies (http://research-pub.gene.com/IMvigor210CoreBiologies)[1].

② Transcriptomic and clinical data of 657 patients from three phase II clinical trials and one phase I clinical trial of atezolizumab were collected from the European Genome-Phenome Archive (EGA) (Study ID EGAS00001004343[2]; https://ega-archive.org/studies/EGAS00001004343). These included 208 patients from a phase II clinical trial in mUC (IMvigor210[3], NCT02108652), 81 patients from a phase II clinical trial in non-small cell lung cancer (NSCLC) (POPLAR[4], NCT01903993), and 162 patients from a phase II clinical trial with atezolizumab or atezolizumab + bevacizumab treatment in renal cell carcinoma (RCC) (IMmotion150[5], NCT01984242), and a total of 206 patients with mUC, NSCLC, or RCC from a phase I basket trial (PCD4989g[6], NCT01375842). Expression matrices of TPM-normalized counts were download and convert to log2(TPM +1).

③ Log2(TPM+1) transformed counts data and matched survival data from 407 patients treated with atezolizumab + bevacizumab from a phase III clinical trial in RCC (IMmotion151, NCT02420821) were collected from the EGA (Study ID EGAS00001004353[7], https://ega-archive.org/studies/EGAS00001004353).

④ Data from 170 patients with advanced clear cell RCC from three clinical trials of nivolumab (anti-PD-1 antibody) were obtained from a report by Braun et al[8]. (https://www.ncbi.nlm.nih.gov/pmc/articles/PMC7499153/bin/NIHMS1611472-supplement-Table_S4__RNA_expression__normalized_expression_matrix_.xlsx). These patients were from CheckMate 009[9](NCT01358721), CheckMate 010[10](NCT01354431), and CheckMate 025[11](NCT01668784).

⑤ Data from 51 patients with advanced melanoma from clinical trials of nivolumab (CA209-038, NCT01621490) were obtained from the Gene Expression Omnibus through the accession number GSE91061 [12](https://www.ncbi.nlm.nih.gov/geo/query/acc.cgi?acc=GSE91061). The transcription profiles (FPKM normalized) of GSE91061 were transformed into TPM.

⑥ RNA-seq data (TPM normalized) of The Cancer Genome Atlas (TCGA)

Pan-Cancer processed on 2016-09-01 were obtained from the University California Santa Cruz Xena browser TCGA hub (https://tcga.xenahubs.net/). Corresponding clinical data updated on 2018-09-13 were downloaded from supplementary data of a published TCGA Pan-Cancer research[13] (https://www.cell.com/cms/10.1016/j.cell.2018.02.052/attachment/bbf46a06-1fb0-417a-a259-fd47591180e4/mmc1.xlsx).

Clinical samples collection

Bladder urothelial carcinoma and kidney renal clear cell carcinoma samples and corresponding adjacent normal tissues were resected from patients hospitalized in the First Affiliated Hospital of Sun Yat-sen University from 2020 to 2022. Patients are between the ages of 18 and 80. All patients were diagnosed through radiological examination and pathology methods. Before surgery, none of the patients received neoadjuvant chemotherapy or radiotherapy. The study was reviewed and approved by the Institutional Ethics Committee for Clinical Research and Animal Trials Ethical of the First Affiliated Hospital of Sun Yat-sen University [(2021)143], [(2021)144]. Written informed consent was obtained from participating patients. All experiments were performed following the ethical standards of the Helsinki Declaration.

Cell line

MB49 cell lines were purchased from Procell(Procell Life Science&Technology Co., Ltd). MB49 was raised in DMEM medium supplemented with 10% fetal bovine serum Cells were cultured in a humidified incubator with 5% CO_2 at 37°C.

Protein expression profile of RPLP0

The difference of RPLP0 protein level between normal and tumor tissues in CPTAC database were explored through The University of Alabama at Birmingham Cancer data analysis Portal (UALCAN) (http://ualcan.path.uab.edu)[14]. Images of immunohistochemistry staining of RPLP0 in tumors and normal tissues were downloaded from the Human Protein Atlas (https://www.proteinatlas.org/).

Supplementary References

1. Mariathasan, S., et al., *TGFbeta attenuates tumour response to PD-L1 blockade by contributing to exclusion of T cells.* Nature, 2018. **554**(7693): p. 544-548.
2. Banchereau, R., et al., *Molecular determinants of response to PD-L1 blockade across tumor types.* Nat Commun, 2021. **12**(1): p. 3969.
3. Balar, A.V., et al., *Atezolizumab as first-line treatment in cisplatin-ineligible patients with locally advanced and metastatic urothelial carcinoma: a single-arm, multicentre, phase 2 trial.* Lancet, 2017. **389**(10064): p. 67-76.
4. Fehrenbacher, L., et al., *Atezolizumab versus docetaxel for patients with previously treated non-small-cell lung cancer (POPLAR): a multicentre, open-label, phase 2 randomised controlled trial.* Lancet, 2016. **387**(10030): p. 1837-46.
5. McDermott, D.F., et al., *Clinical activity and molecular correlates of response to atezolizumab alone or in combination with bevacizumab versus sunitinib in renal cell*

carcinoma. Nat Med, 2018. **24**(6): p. 749-757.

6. Herbst, R.S., et al., *Predictive correlates of response to the anti-PD-L1 antibody MPDL3280A in cancer patients.* Nature, 2014. **515**(7528): p. 563-7.
7. Rini, B.I., et al., *Atezolizumab plus bevacizumab versus sunitinib in patients with previously untreated metastatic renal cell carcinoma (IMmotion151): a multicentre, open-label, phase 3, randomised controlled trial.* Lancet, 2019. **393**(10189): p. 2404-2415.
8. Braun, D.A., et al., *Interplay of somatic alterations and immune infiltration modulates response to PD-1 blockade in advanced clear cell renal cell carcinoma.* Nat Med, 2020. **26**(6): p. 909-918.
9. Choueiri, T.K., et al., *Immunomodulatory Activity of Nivolumab in Metastatic Renal Cell Carcinoma.* Clin Cancer Res, 2016. **22**(22): p. 5461-5471.
10. Motzer, R.J., et al., *Nivolumab for Metastatic Renal Cell Carcinoma: Results of a Randomized Phase II Trial.* J Clin Oncol, 2015. **33**(13): p. 1430-7.
11. Motzer, R.J., et al., *Nivolumab plus Ipilimumab versus Sunitinib in Advanced Renal-Cell Carcinoma.* N Engl J Med, 2018. **378**(14): p. 1277-1290.
12. Riaz, N., et al., *Tumor and Microenvironment Evolution during Immunotherapy with Nivolumab.* Cell, 2017. **171**(4): p. 934-949 e16.
13. Liu, J., et al., *An Integrated TCGA Pan-Cancer Clinical Data Resource to Drive High-Quality Survival Outcome Analytics.* Cell, 2018. **173**(2).
14. Chandrashekar, D.S., et al., *UALCAN: An update to the integrated cancer data analysis platform.* Neoplasia (New York, N.Y.), 2022. **25**: p. 18-27.
