# Supplementary material for: Interplay between tumor mutation burden and the tumor microenvironment predicts the prognosis of pan-cancer anti-PD-1/PD-L1 therapy
Source: Front Immunol. 2025 Jul 24;16:1557461. doi: 10.3389/fimmu.2025.1557461 (PMC12328289; doi:10.3389/fimmu.2025.1557461)
Supplement: Supplementary file 3 [file DataSheet3.docx]

**Supplementary Method**

**Genomic and clinical data sources and preprocessing**

RNA-seq data obtained before immunotherapy and matched clinical data of patients who received anti-PD1/PDL1 immunotherapy in several cohorts across multiple cancer types and other public data analyzed in this work were collected.

1. RNA-seq data in Transcripts Per Million (TPM) and matched clinical data of patients with metastatic urothelial cancer (mUC) treated with atezolizumab (anti-PD-L1 antibody) were retrieved from the R package IMvigor210CoreBiologies (<http://research-pub.gene.com/IMvigor210CoreBiologies>)[1].
2. Transcriptomic and clinical data of 657 patients from three phase II clinical trials and one phase I clinical trial of atezolizumab were collected from the European Genome-Phenome Archive (EGA) (Study ID EGAS00001004343[2]; <https://ega-archive.org/studies/EGAS00001004343>). These included 208 patients from a phase II clinical trial in mUC (IMvigor210[3], NCT02108652), 81 patients from a phase II clinical trial in non-small cell lung cancer (NSCLC) (POPLAR[4], NCT01903993), and 162 patients from a phase II clinical trial with atezolizumab or atezolizumab + bevacizumab treatment in renal cell carcinoma (RCC) (IMmotion150[5], NCT01984242), and a total of 206 patients with mUC, NSCLC, or RCC from a phase I basket trial (PCD4989g[6], NCT01375842). Expression matrices of TPM-normalized counts were download and convert to log2(TPM +1).
3. Log2(TPM+1) transformed counts data and matched survival data from 407 patients treated with atezolizumab + bevacizumab from a phase III clinical trial in RCC (IMmotion151, NCT02420821) were collected from the EGA (Study ID EGAS00001004353[7], <https://ega-archive.org/studies/EGAS00001004353>).
4. Data from 170 patients with advanced clear cell RCC from three clinical trials of nivolumab (anti-PD-1 antibody) were obtained from a report by Braun et al[8]. (<https://www.ncbi.nlm.nih.gov/pmc/articles/PMC7499153/bin/NIHMS1611472-supplement-Table_S4__RNA_expression__normalized_expression_matrix_.xlsx>). These patients were from CheckMate 009[9](NCT01358721), CheckMate 010[10](NCT01354431), and CheckMate 025[11](NCT01668784).
5. Data from 51 patients with advanced melanoma from clinical trials of nivolumab (CA209-038, NCT01621490) were obtained from the Gene Expression Omnibus through the accession number GSE91061 [12](<https://www.ncbi.nlm.nih.gov/geo/query/acc.cgi?acc=GSE91061>). The transcription profiles (FPKM normalized) of GSE91061 were transformed into TPM.
6. RNA-seq data (TPM normalized) of The Cancer Genome Atlas (TCGA) Pan-Cancer processed on 2016-09-01 were obtained from the University California Santa Cruz Xena browser TCGA hub (<https://tcga.xenahubs.net/>). Corresponding clinical data updated on 2018-09-13 were downloaded from supplementary data of a published TCGA Pan-Cancer research[13] (<https://www.cell.com/cms/10.1016/j.cell.2018.02.052/attachment/bbf46a06-1fb0-417a-a259-fd47591180e4/mmc1.xlsx>).

**Clinical samples collection**

Bladder urothelial carcinoma and kidney renal clear cell carcinoma samples and corresponding adjacent normal tissues were resected from patients hospitalized in the First Affiliated Hospital of Sun Yat-sen University from 2020 to 2022. Patients are between the ages of 18 and 80. All patients were diagnosed through radiological examination and pathology methods. Before surgery, none of the patients received neoadjuvant chemotherapy or radiotherapy. The study was reviewed and approved by the Institutional Ethics Committee for Clinical Research and Animal Trials Ethical of the First Affiliated Hospital of Sun Yat-sen University [(2021)143], [(2021)144]. Written informed consent was obtained from participating patients. All experiments were performed following the ethical standards of the Helsinki Declaration.

**Cell line**

MB49 cell lines were purchased from Procell(Procell Life Science&Technology Co., Ltd). MB49 was raised in DMEM medium supplemented with 10% fetal bovine serum Cells were cultured in a humidified incubator with 5% CO2 at 37°C.

**Protein expression profile of RPLP0**

The difference of RPLP0 protein level between normal and tumor tissues in CPTAC database were explored through The University of Alabama at Birmingham Cancer data analysis Portal (UALCAN) (<http://ualcan.path.uab.edu>)[14]. Images of immunohistochemistry staining of RPLP0 in tumors and normal tissues were downloaded from the Human Protein Atlas (<https://www.proteinatlas.org/>).

**Supplementary References**

1. Mariathasan, S., et al., *TGFbeta attenuates tumour response to PD-L1 blockade by contributing to exclusion of T cells.* Nature, 2018. **554**(7693): p. 544-548.

2. Banchereau, R., et al., *Molecular determinants of response to PD-L1 blockade across tumor types.* Nat Commun, 2021. **12**(1): p. 3969.

3. Balar, A.V., et al., *Atezolizumab as first-line treatment in cisplatin-ineligible patients with locally advanced and metastatic urothelial carcinoma: a single-arm, multicentre, phase 2 trial.* Lancet, 2017. **389**(10064): p. 67-76.

4. Fehrenbacher, L., et al., *Atezolizumab versus docetaxel for patients with previously treated non-small-cell lung cancer (POPLAR): a multicentre, open-label, phase 2 randomised controlled trial.* Lancet, 2016. **387**(10030): p. 1837-46.

5. McDermott, D.F., et al., *Clinical activity and molecular correlates of response to atezolizumab alone or in combination with bevacizumab versus sunitinib in renal cell carcinoma.* Nat Med, 2018. **24**(6): p. 749-757.

6. Herbst, R.S., et al., *Predictive correlates of response to the anti-PD-L1 antibody MPDL3280A in cancer patients.* Nature, 2014. **515**(7528): p. 563-7.

7. Rini, B.I., et al., *Atezolizumab plus bevacizumab versus sunitinib in patients with previously untreated metastatic renal cell carcinoma (IMmotion151): a multicentre, open-label, phase 3, randomised controlled trial.* Lancet, 2019. **393**(10189): p. 2404-2415.

8. Braun, D.A., et al., *Interplay of somatic alterations and immune infiltration modulates response to PD-1 blockade in advanced clear cell renal cell carcinoma.* Nat Med, 2020. **26**(6): p. 909-918.

9. Choueiri, T.K., et al., *Immunomodulatory Activity of Nivolumab in Metastatic Renal Cell Carcinoma.* Clin Cancer Res, 2016. **22**(22): p. 5461-5471.

10. Motzer, R.J., et al., *Nivolumab for Metastatic Renal Cell Carcinoma: Results of a Randomized Phase II Trial.* J Clin Oncol, 2015. **33**(13): p. 1430-7.

11. Motzer, R.J., et al., *Nivolumab plus Ipilimumab versus Sunitinib in Advanced Renal-Cell Carcinoma.* N Engl J Med, 2018. **378**(14): p. 1277-1290.

12. Riaz, N., et al., *Tumor and Microenvironment Evolution during Immunotherapy with Nivolumab.* Cell, 2017. **171**(4): p. 934-949 e16.

13. Liu, J., et al., *An Integrated TCGA Pan-Cancer Clinical Data Resource to Drive High-Quality Survival Outcome Analytics.* Cell, 2018. **173**(2).

14. Chandrashekar, D.S., et al., *UALCAN: An update to the integrated cancer data analysis platform.* Neoplasia (New York, N.Y.), 2022. **25**: p. 18-27.
