# Supplementary figures and images for: Interplay between tumor mutation burden and the tumor microenvironment predicts the prognosis of pan-cancer anti-PD-1/PD-L1 therapy
Source: Front Immunol. 2025 Jul 24;16:1557461. doi: 10.3389/fimmu.2025.1557461 (PMC12328289; doi:10.3389/fimmu.2025.1557461)

Figure7C

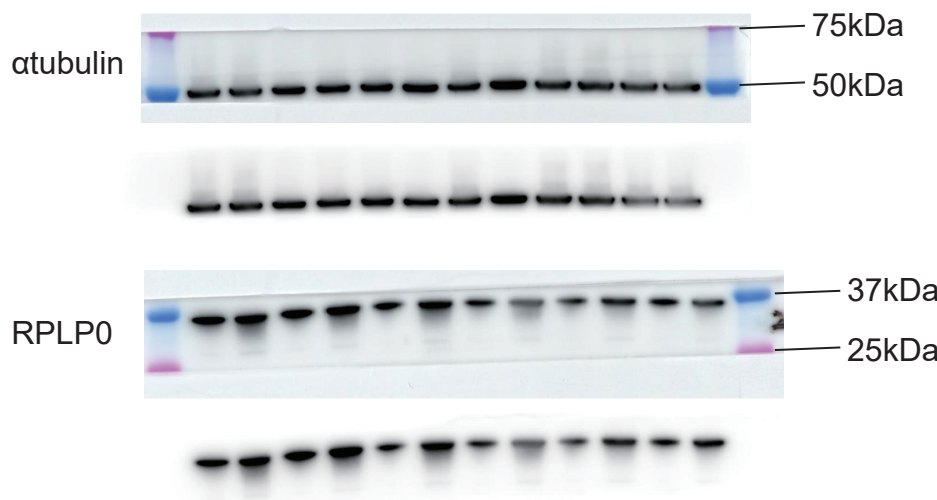

Figure7D

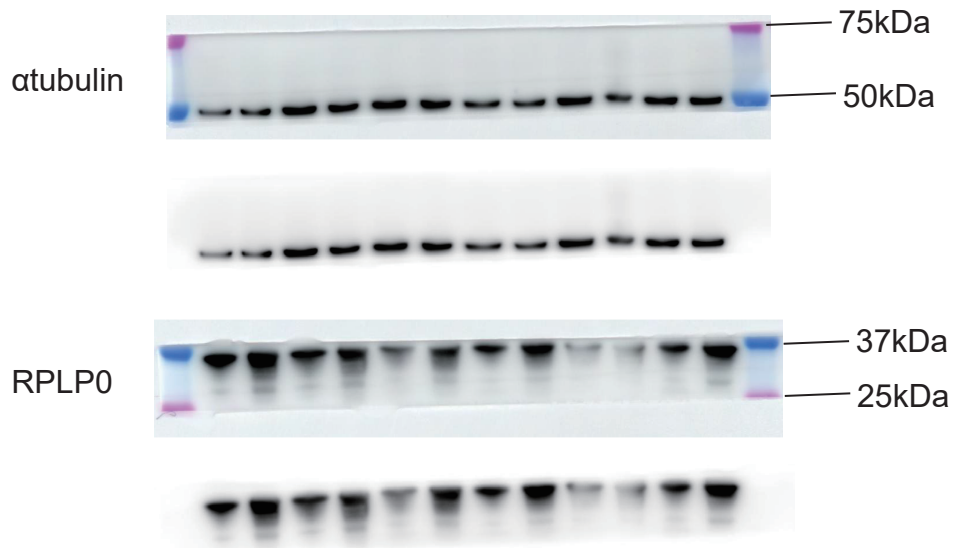

Supplement: Supplementary file 4 [file DataSheet4.pdf]

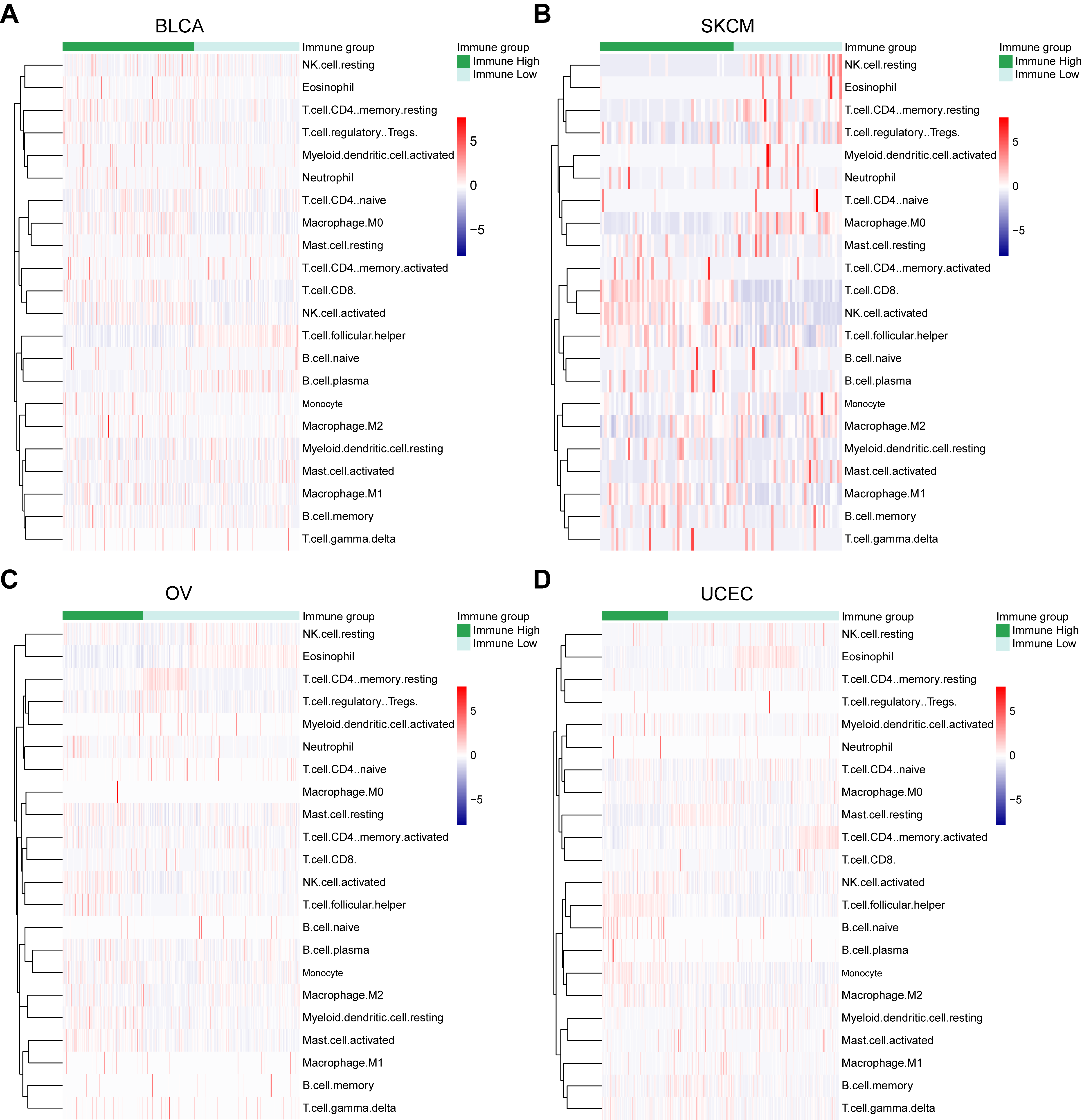

Supplement: Supplementary file 6 [file Image1.tif]

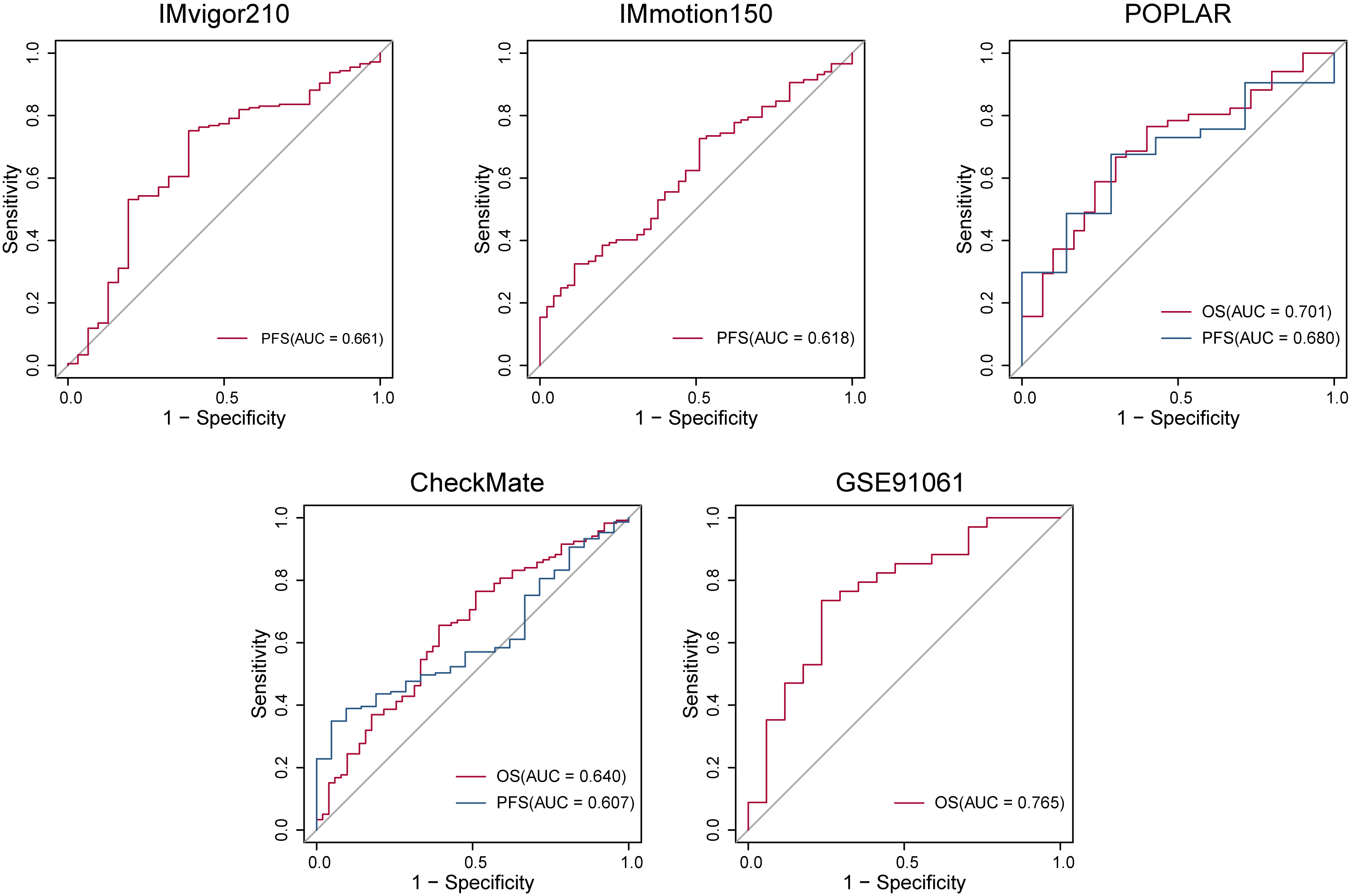

Supplement: Supplementary file 7 [file Image2.tif]

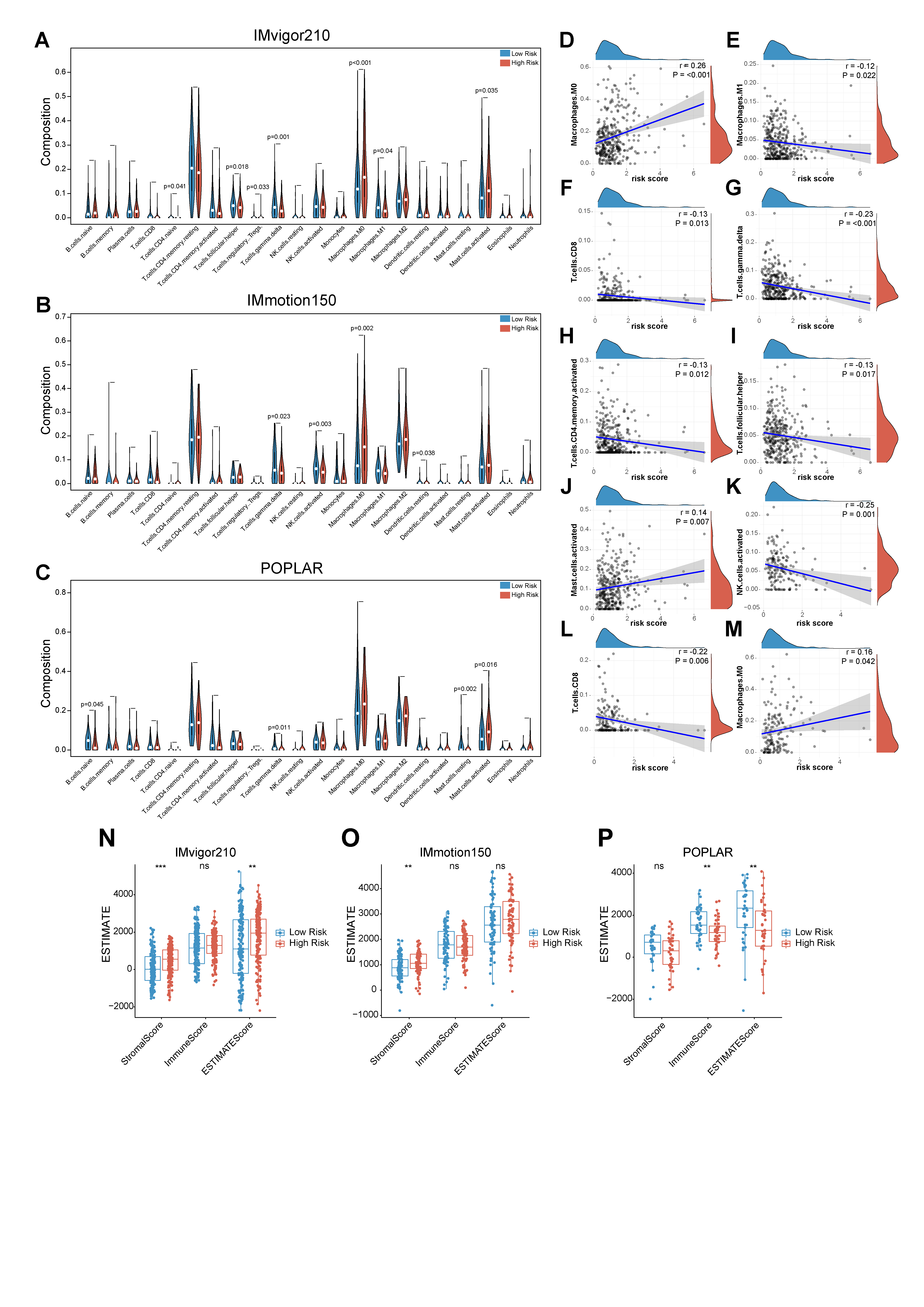

Supplement: Supplementary file 8 [file Image3.tif]

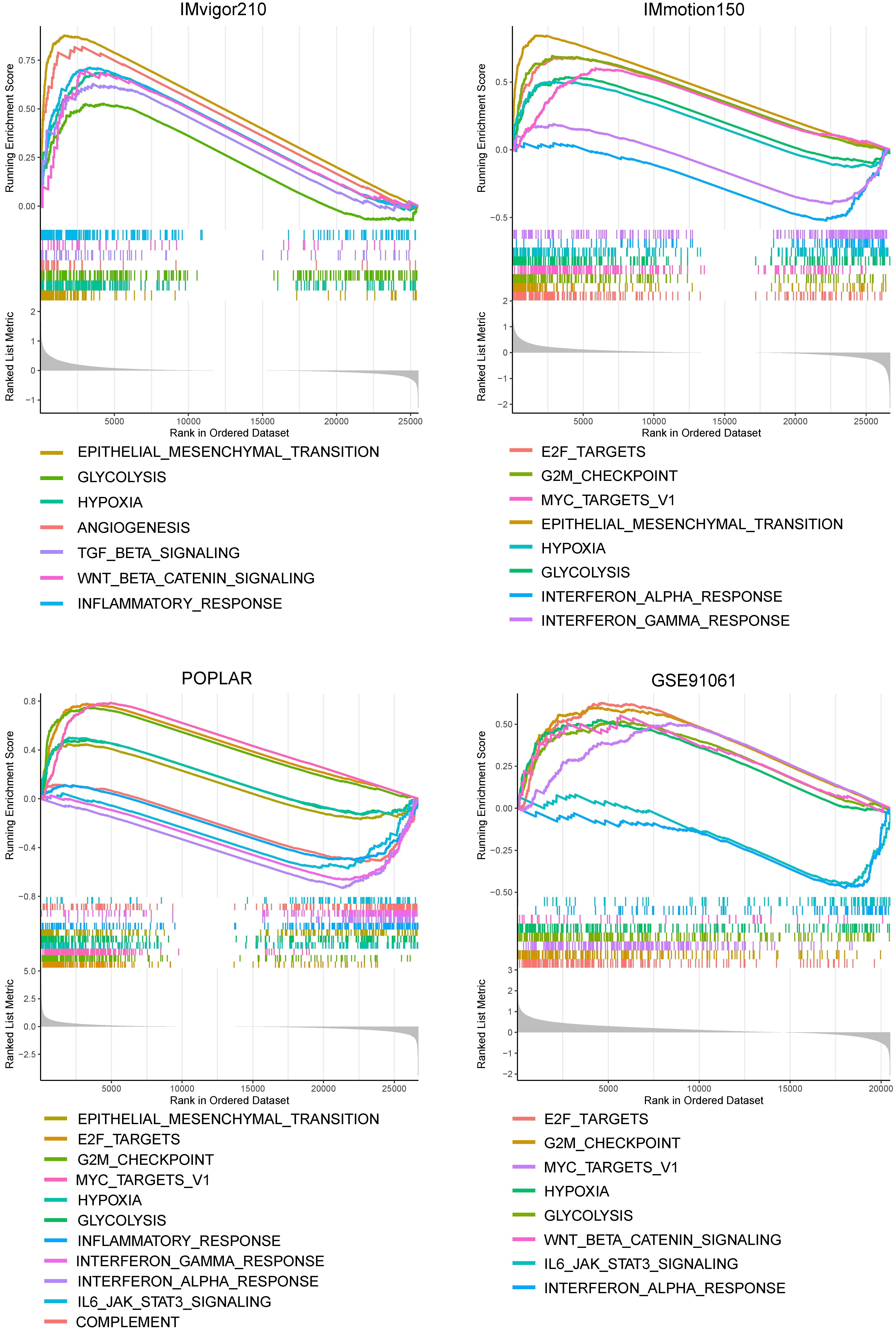

Supplement: Supplementary file 9 [file Image4.tif]

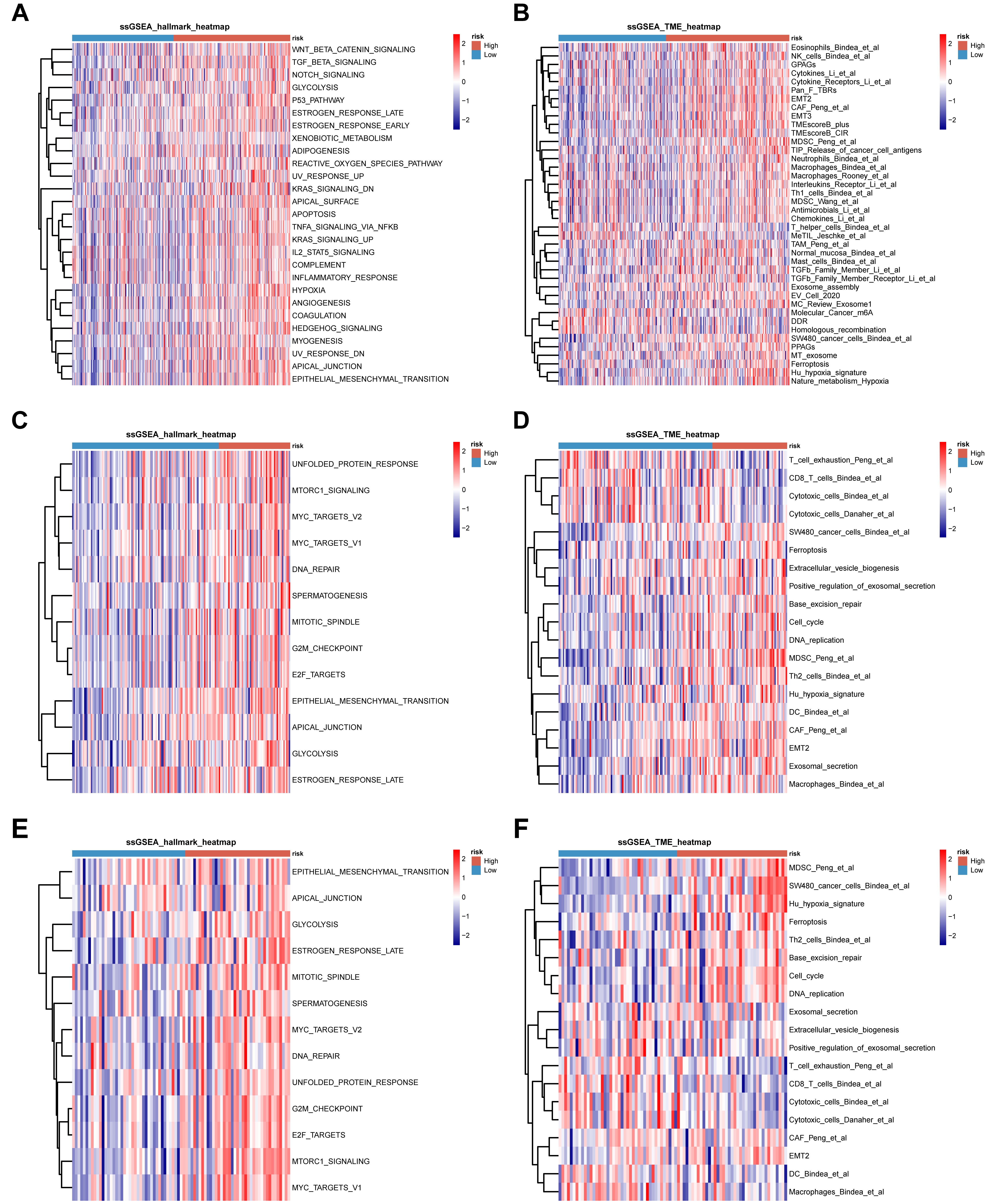

Supplement: Supplementary file 10 [file Image5.tif]

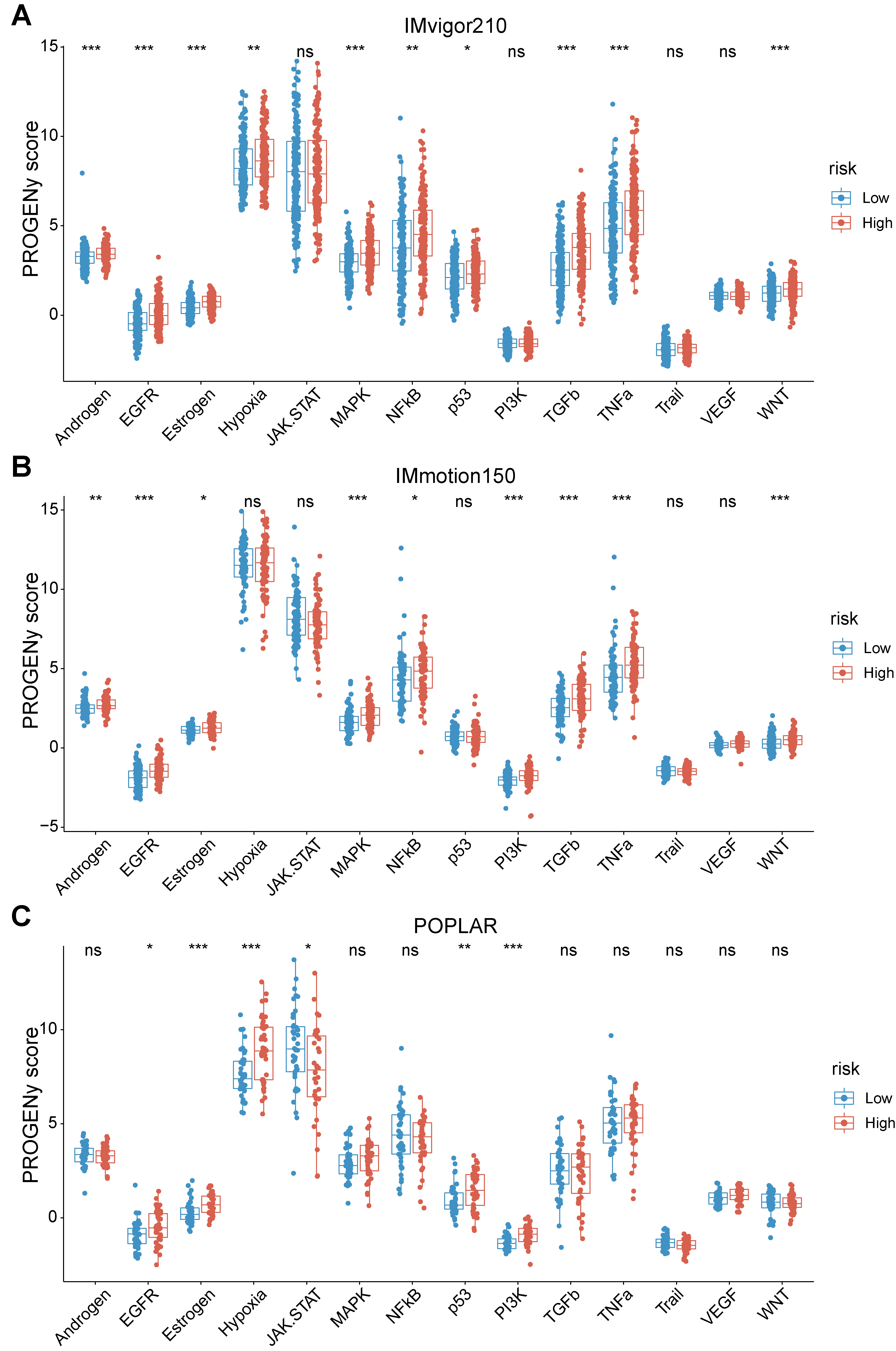

Supplement: Supplementary file 11 [file Image6.tif]

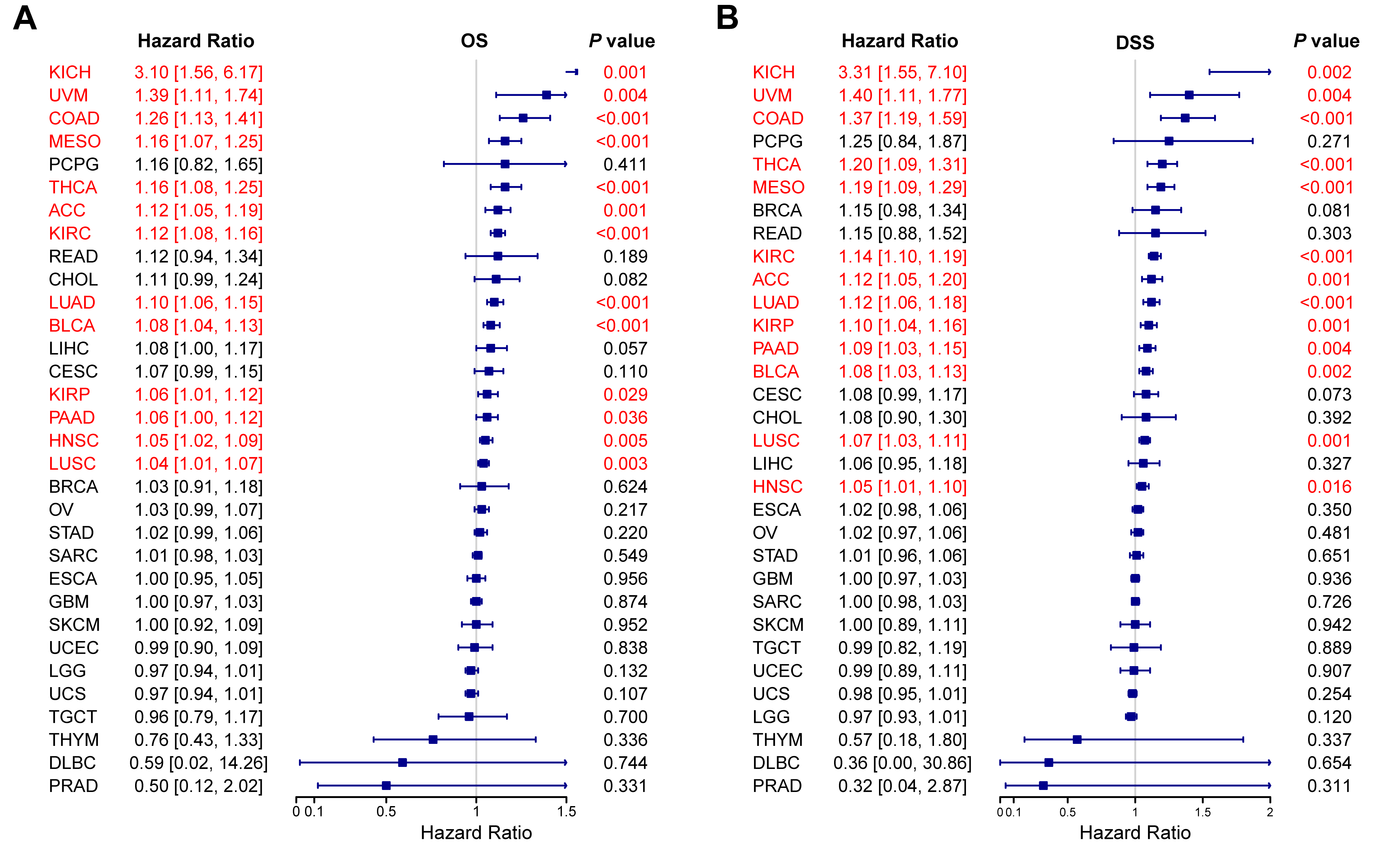

Supplement: Supplementary file 12 [file Image7.tif]

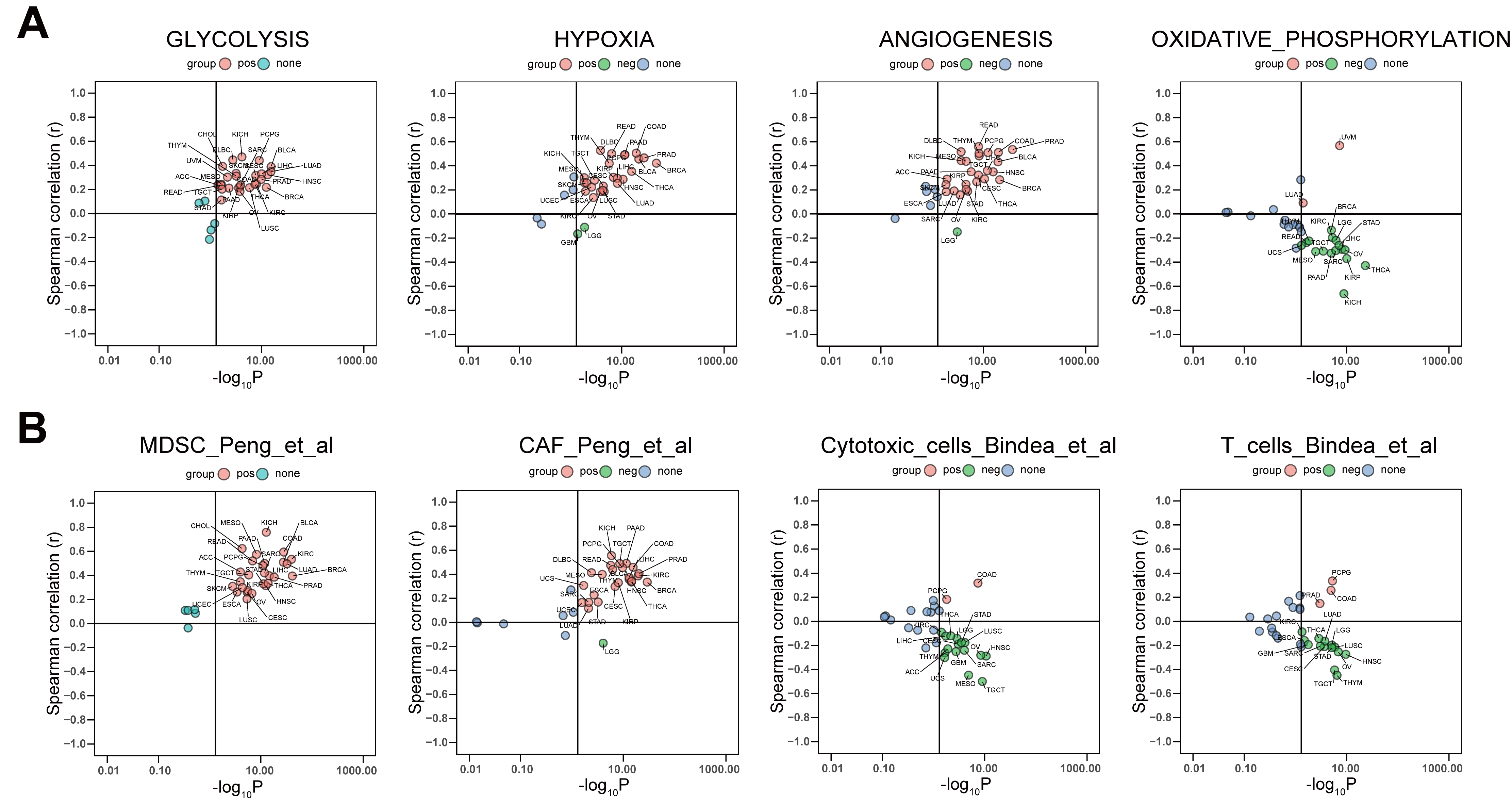

Supplement: Supplementary file 13 [file Image8.tif]

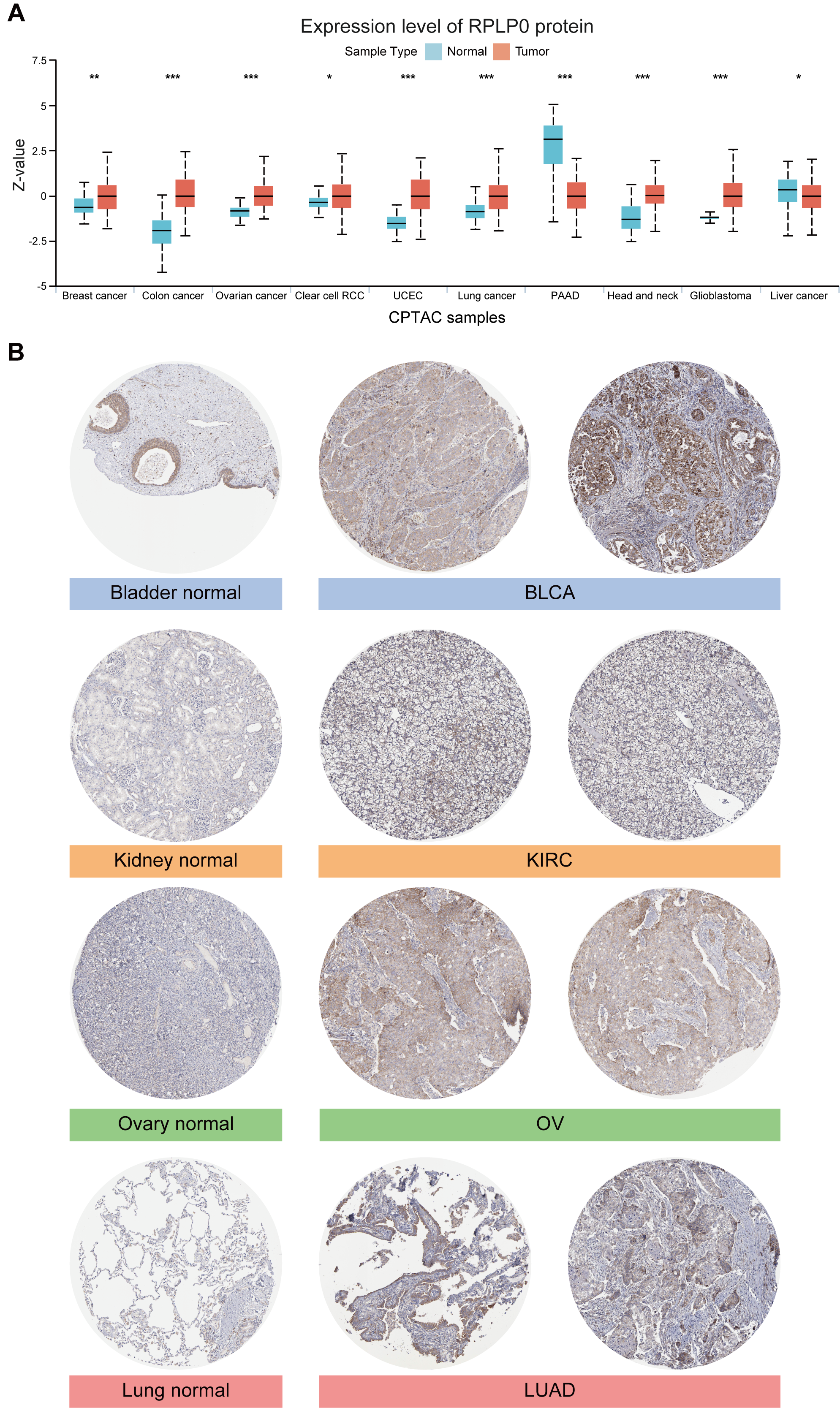

Supplement: Supplementary file 14 [file Image9.tif]

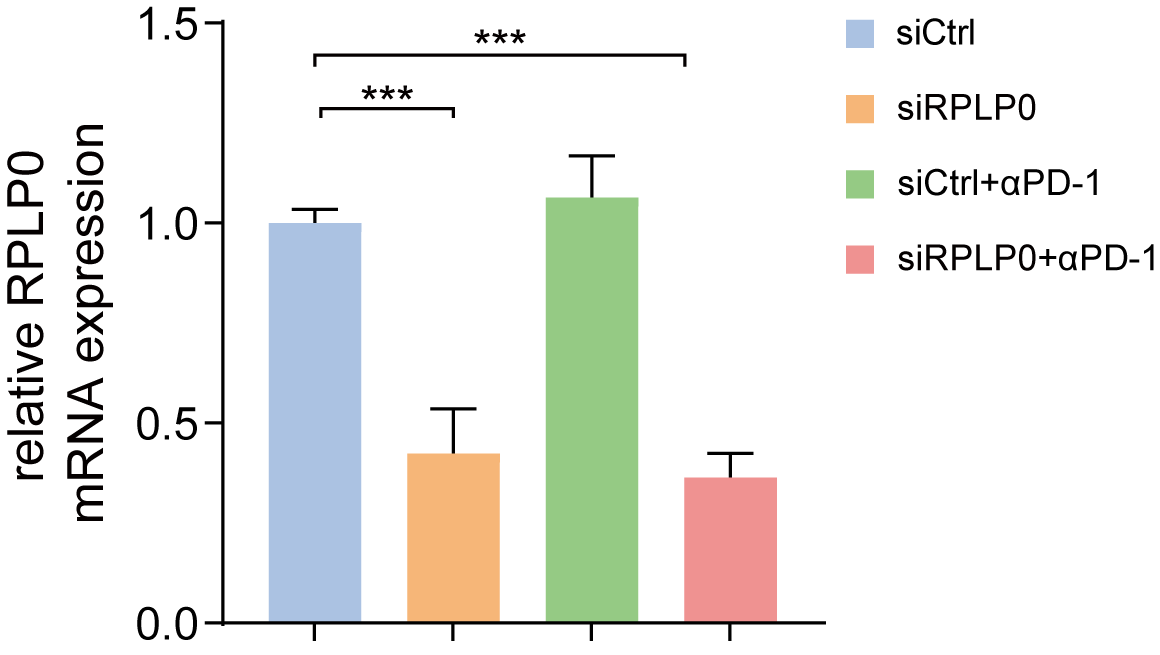

Supplement: Supplementary file 15 [file Image10.tif]
